# The Role of the N170 in Linking Stimuli to Feedback—Effects of Stimulus Modality and Feedback Delay

**DOI:** 10.1111/psyp.70050

**Published:** 2025-04-15

**Authors:** Madita Röhlinger, Christine Albrecht, Christian Bellebaum

**Affiliations:** ^1^ Institute for Experimental Psychology, Faculty of Mathematics and Natural Sciences Heinrich Heine University Düsseldorf Düsseldorf Germany

**Keywords:** feedback learning, feedback timing, FRN/RewP, N170, sensory reactivation, stimulus modality

## Abstract

With increasing feedback delay, feedback processing appears to shift from the striatum to the hippocampus. In addition, higher‐order sensory areas might be involved in bridging a temporal gap between stimulus and feedback by reactivating the representation of the feedback‐predicting stimulus during feedback processing. We hypothesized that the feedback‐locked N170, an occipito‐temporal event‐related potential (ERP) component linked to higher‐order visual processing, is more pronounced when delayed feedback is provided for choices between visual compared to auditory stimuli. 35 subjects completed a probabilistic feedback learning task with immediate (1 s) and delayed (7 s) monetary feedback for choices between visual or auditory stimuli. Participants successfully learned to choose the more rewarding stimuli irrespective of stimulus modality. For the N170 amplitude over the right hemisphere, we found an interaction between feedback timing and the modality of the chosen stimulus. Only for delayed feedback, the N170 was more pronounced for choices between visual than auditory stimuli. Moreover, in this condition, the N170 amplitude particularly reflected the reward prediction error (PE), with larger amplitudes for positive PEs and lower amplitudes for negative PEs. This suggests that the N170 reflects feedback‐locked reactivations in higher‐order visual areas mediated by the reward PE. While these effects need to be studied further, we discuss the N170 as a counterpart to the feedback‐related negativity (FRN) regarding interacting influences of feedback valence, feedback timing, and PE.

## Impact Statement

How can our brain draw a connection between the decisions we make and the feedback we receive? With the present work, we demonstrate that the sensory reactivation of a chosen stimulus’ representation in visual areas, reflected in the N170, could establish an association between the selection of a stimulus and temporally delayed feedback.

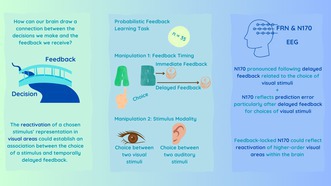

## 1 Introduction

Numerous studies underpin the involvement of a dopaminergic, striatal, mesocorticolimbic reward system in processing performance feedback, that is, when human study participants receive positive or negative outcomes for their choice actions (for reviews, see Delgado 2007; Haber and Knutson 2010; Wang et al. 2016). However, neural mechanisms involved in feedback processing are affected by the temporal proximity of an action and its outcome (Jocham et al. 2016). A study by Foerde and Shohamy (2011) underlined the role of striatal activity in processing immediate feedback but found pronounced hippocampal activity in processing delayed feedback (after a couple of seconds). Causal inferences concerning the neural mechanisms of processing immediate and delayed feedback could be drawn from lesion studies: Parkinson's disease patients suffering from striatal dysfunctions (Damier et al. 1999) had problems learning from immediate, but not from delayed feedback (Foerde and Shohamy 2011). Conversely, amnestic patients with presumed lesions in the medial temporal lobe (MTL) including the hippocampus had problems learning from delayed, but not from immediate feedback (Foerde et al. 2013). Staresina and Davachi (2009) suggested that the role of the hippocampus is to bind representations separated by space and time to bridge gaps in our experience. Several researchers have suggested that, in the absence of immediate feedback to the striatum, the MTL may be recruited to bind an individual's response with the delayed feedback, despite their separation in time (Arbel et al. 2017; Foerde et al. 2013; Peterburs et al. 2016).

In studies assessing neural feedback processing by means of electroencephalography (EEG), delays have been found to differentially affect two event‐related potential (ERP) components that have been associated with the reward system and the MTL, respectively (Arbel et al. 2017; Höltje and Mecklinger 2020; Kim and Arbel 2019; Peterburs et al. 2016). The feedback‐related negativity (FRN) peaks around 250 to 300 ms after feedback presentation at frontocentral electrode sites and is more pronounced for negative than positive feedback (Becker et al. 2014; Bellebaum and Daum 2008; Foti et al. 2011; Holroyd and Coles 2002; Miltner et al. 1997; Nieuwenhuis et al. 2004), possibly because a positive component referred to as Reward Positivity (RewP; for a review see Proudfit 2015) drives the signal toward positive amplitudes for positive feedback. The amplitude of the signal in the FRN/RewP time window reflects a prediction error (PE) that indicates whether feedback is better or worse than expected (Burnside et al. 2019; Fischer and Ullsperger 2013; Kirsch et al. 2022; Sambrook and Goslin 2015; Weber and Bellebaum 2024). A PE is encoded by midbrain dopaminergic neurons, for instance in the substantia nigra (Schultz et al. 1997; Zaghloul et al. 2009), suggesting that the FRN indirectly reflects activity of the midbrain dopamine system (Foti et al. 2015; Hauser et al. 2014; Holroyd and Coles 2002). Williams et al. (2020) provide evidence that the FRN reflects an underlying learning process that drives behavioral adaptation based on PEs. Having been linked to striatal activity (Becker et al. 2014; Carlson et al. 2011; Foti et al. 2011), and thus a dopamine projection site (Chuhma et al. 2023; Oldehinkel et al. 2022; Zhang et al. 2015), the FRN difference wave for negative –positive feedback better differentiates feedback valence when feedback is presented immediately (Arbel et al. 2017; Höltje and Mecklinger 2020; Peterburs et al. 2016; Weinberg et al. 2012; Weismüller and Bellebaum 2016). Evidence suggests, however, that the FRN is not directly generated by the striatum (Cohen et al. 2011), but by the medial prefrontal cortex, more specifically, the anterior cingulate cortex (Nieuwenhuis et al. 2005; Hauser et al. 2014; Becker et al. 2014; Oerlemans et al. 2025), which in turn receives projections from the striatum (Chau et al. 2018; Hauser et al. 2014).

In contrast, the N170, a negative deflection about 170 ms after visual stimulus presentation at lateral temporal electrode sites (Bentin et al. 1996), was repeatedly found to be more pronounced for delayed than immediate feedback (Arbel et al. 2017; Höltje and Mecklinger 2020; Kim and Arbel 2019; but see Albrecht et al. 2023, for the opposite pattern). Arbel et al. (2017) and Kim and Arbel (2019) hypothesized that the N170 is generated by a delayed reward signal to reinforce a memory representation of a stimulus stored in the MTL. In this line, Baker and Holroyd (2009) demonstrated that the spatial location of feedback stimuli elicited a pronounced N170 response associated with right MTL activation in a navigational feedback learning task. In subsequent studies, Baker and Holroyd (2013) and Baker et al. (2015) localized the N170 in this task to the right parahippocampal region, proposing that the parahippocampal cortex encodes salient information essential for spatial navigation.

With the present work we aim to investigate an alternative explanation regarding larger N170 amplitudes for delayed feedback: The N170 is usually investigated in the context of higher visual processing, being particularly pronounced for faces (Bentin et al. 1996; Itier and Taylor 2004; for a review see Yovel 2016) and words (for a review see Carreiras et al. 2014), but also cars (Kloth et al. 2013). For faces and words, an origin in the fusiform gyrus was found (Brem et al. 2006; Deffke et al. 2007; Gao et al. 2019; Iidaka et al. 2006), which contains specialized regions for diverse stimulus categories (Cohen et al. 2002; Kanwisher et al. 1997; for an overview see Weiner and Zilles 2016). Thus, a pronounced N170 after delayed feedback may indicate the activation of higher‐order visual areas during the processing of (delayed) feedback, possibly mediated by the MTL.

If feedback is delayed, a reactivation of the representations of the associated stimulus might be the mechanism to bridge the temporal gap between stimulus and feedback. Support for this assumption comes from several fMRI studies: For example, participants in a study by Pleger et al. (2008) had to discriminate somatosensory stimuli regarding their frequency (high vs. low) and were rewarded for correct judgments. Notably, the primary somatosensory cortex was reactivated when reward was presented, an effect mediated by dopamine (Pleger et al. 2009). In a study by Schiffer et al. (2014), reward activated stimulus‐category‐specific representations of reward‐associated stimuli in visual association cortices.

In the present study, we want to examine whether the N170 for delayed feedback represents a reactivation of a previously selected visual stimulus to bridge the temporal gap and assign credit to the stimulus. To test this, we manipulate the modality of the stimuli between which participants have to choose in a feedback learning task. More specifically, participants receive visual feedback for choices between two visual or two auditory stimuli. We hypothesize that the N170 has a larger amplitude when the feedback is associated with visual than with auditory stimuli and that this effect is stronger for delayed compared to immediate feedback. Given that the right hemisphere plays a dominant role in processing certain visual stimuli, such as faces (Rossion 2014), and in N170 generation in different contexts (Baker and Holroyd 2009; Baker and Holroyd 2013; Baker et al. 2015; Kim and Arbel 2019), we were particularly interested in whether the effects would be stronger over the right hemisphere. In addition, we explore whether the PE is represented in the N170, possibly depending on stimulus modality, feedback timing, and hemisphere. For this purpose, we will model trial‐by‐trial fluctuations of the PE using the behavioral learning data. Given that the hippocampus shows PE‐related activity (Dickerson et al. 2011) and that the N170 may be mediated by MTL processing, it is conceivable that the PE is reflected in the N170 amplitude, especially following delayed feedback for the choice between visual stimuli and over the right hemisphere. Regarding the FRN, we aimed to replicate previous effects for PE coding and effects of the timing of feedback and explore effects of the modality of the stimulus associated with the feedback in interaction with these factors, without a specific hypothesis.

## 2 Method

### 2.1 Participants

The sample size was planned a priori and based on the number of participants in previous studies investigating the effects of feedback timing on FRN and N170: Arbel et al. (2017) found a significant effect of feedback timing on the N170 in a study with 21 subjects. In the planned study, we were particularly interested in the interaction between feedback timing and stimulus modality and also in higher‐order interactions (see Data Analysis for details), which suggests that a larger sample size was needed to reach adequate power. We thus preregistered to recruit 40 healthy young adults (18–40 years) for participation in the experiment. Exclusion criteria were a history of neurological or psychiatric disorders, the regular or acute consumption of substances affecting the central nervous system, knowledge about Hiragana‐Characters, uncorrected impaired vision, and impaired hearing. Of 40 acquired participants, we excluded five participants, three of them because they fulfilled at least one of our exclusion criteria, one because of bad EEG data quality due to alpha waves, and one due to technical problems. The final sample included in the analyses thus consisted of 35 participants, 30 women and 5 men, 2 left‐handed and 33 right‐handed. The mean age was 23.2 years (SD = 4.5 years, Min = 19 years, Max = 35 years).

### 2.2 Experimental Task and Conditions

**FIGURE 1:**
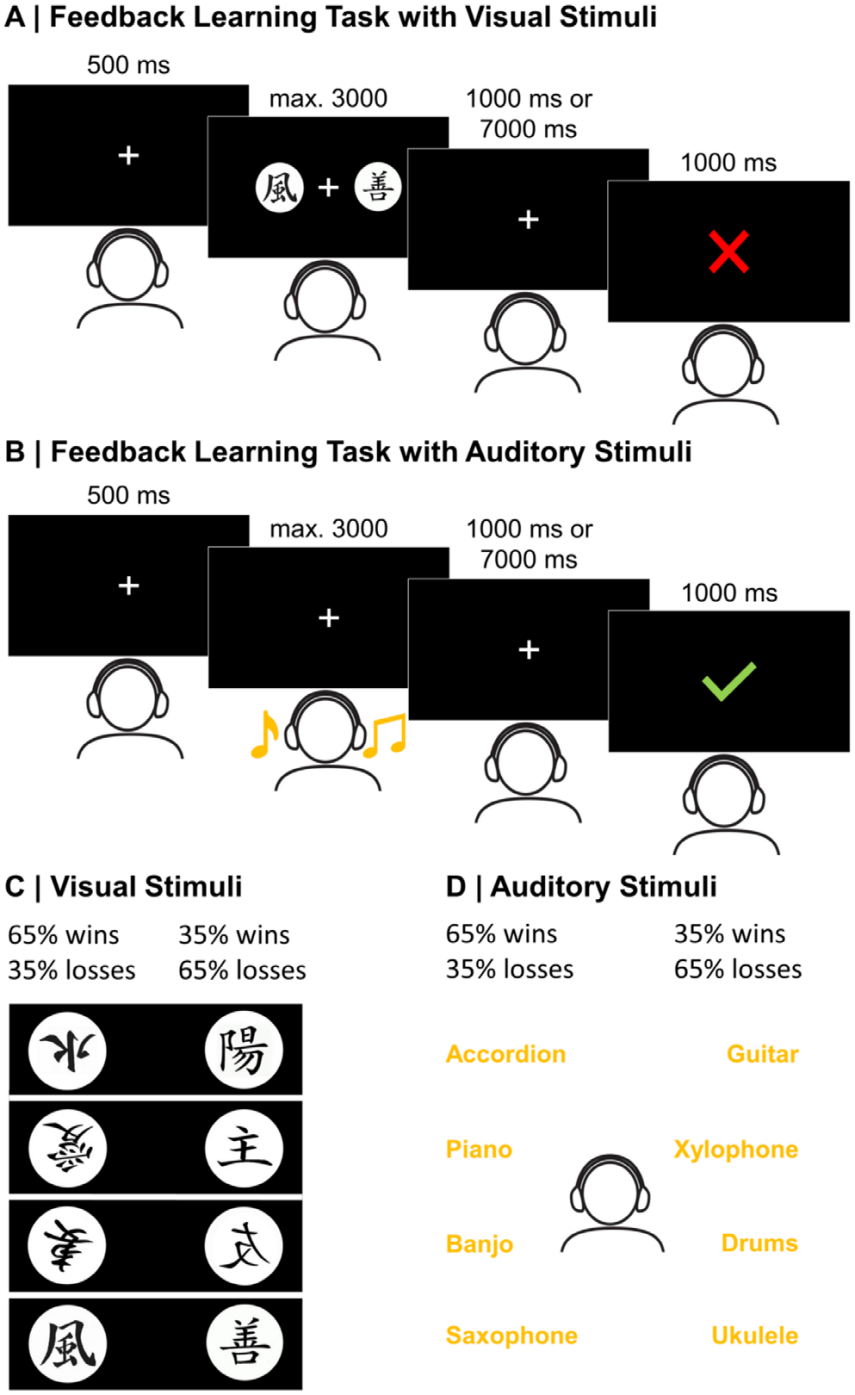
Stimuli and time course of the probabilistic feedback learning tasks. Participants were instructed that the red cross represented a loss of −2 ct while the green tick represented a gain of +4 ct. (A) Feedback learning task with visual stimuli: The assignment of visual stimuli to the left and right sides of the screen was counterbalanced. In this way, feedback could clearly be associated with a stimulus and not with a response side. (B) Feedback learning task with auditory stimuli: The assignment of auditory stimuli to the left and right ears was counterbalanced. In this way, feedback could clearly be associated with a stimulus and not with a response side. (C) Visual stimuli: The neighboring stimuli form the four pairs used for all participants. The more rewarding stimulus (65% wins, 35% losses) was determined randomly when a new stimulus pair was presented. (D) Auditory stimuli: The neighboring stimuli form the four pairs used for all participants. The more rewarding stimulus (65% wins, 35% losses) was determined randomly when a new stimulus pair was presented.

Participants underwent a probabilistic feedback learning task, in which they could learn associations between stimuli and positive or negative monetary feedback (feedback valence: +4 ct vs. −2 ct). The task comprised the two within‐subject factors Stimulus Modality and Feedback Timing: On every trial, each participant could choose between two stimuli. In half of the trials of the experiment, the choice was between two visual stimuli; in the other half of the trials, the choice was between two auditory stimuli (factor Stimulus Modality). Figure 1A shows an exemplary trial for the choice between visual and Figure 1B for the choice between auditory stimuli. Furthermore, feedback appeared 1 s (immediate feedback) or 7 s (delayed feedback) after participants' choice and was always presented visually on the screen (factor Feedback Timing). Participants completed four learning phases with stimuli of one modality (either visual or auditory) before switching to stimuli of the other modality, again for four learning phases, with the order of modalities counterbalanced across participants. In each learning phase, a new stimulus pair was presented, and there were thus eight stimulus pairs in total, four visual and four auditory pairs. Feedback timing (immediate or delayed) remained consistent throughout the phase. The feedback timing changed only at the beginning of a new learning phase, coinciding with the presentation of a new stimulus pair. Thus, feedback timing varied across phases, with the starting condition counterbalanced across participants. Each learning phase consisted of 80 trials and was further divided into 4 blocks of 20 trials. Overall, each participant thus completed 640 trials.

In the visual condition, in every trial a pair of visual stimuli was presented on screen for maximally 3000 ms, one on the left and one on the right side of a centrally presented fixation cross. As stimuli, we used Hiragana‐like characters (see Figure 1C) that cannot easily be verbalized (see Frank et al. 2004). Participants could choose one of the two stimuli by pressing the corresponding (left vs. right) control key on a computer keyboard. The assignment of visual stimuli to the left and right side of the screen was counterbalanced. In this way, feedback could clearly be associated with a stimulus and not with a response side.

In the auditory condition, a pair of auditory stimuli was presented simultaneously via headphones for maximally 3000 ms, one to the left and one to the right ear, while participants' eyes rested on a fixation cross on the screen. As stimuli, we used different melodies played by different instruments to increase distinctiveness (see Figure 1D and listen to an example https://tinyurl.com/mrxtjvt2). Auditory stimuli were downloaded from Pixabay (https://pixabay.com/) and edited with Audacity (https://www.audacityteam.org/). Participants could choose one of the two stimuli by pressing the corresponding (left vs. right) control key on a computer keyboard. The assignment of auditory stimuli to the left and right ear was counterbalanced. In this way, feedback could clearly be associated with a stimulus and not with a response side.

After their choice, feedback was presented. Unbeknown to the participants, one stimulus of each pair was associated with reward in 65% of the trials and with punishment in 35%, while probabilities were reversed for the other stimulus. We chose these contingencies to prevent ceiling effects, as learning with just one stimulus pair at a time in an 80‐trial learning phase might be too easy with higher contingencies. Additionally, these contingencies ensured relatively balanced frequencies of positive and negative feedback, minimizing the risk that one type of feedback would elicit different ERPs simply due to its lower occurrence frequency. The participants' task was to learn which stimulus was more likely to be rewarded and thereby maximize reward through their choices. Both wins and losses contributed to the overall sum of money.

### 2.3 Procedure and Data Acquisition

Upon arrival in the laboratory, participants were informed about the experimental procedure and gave written informed consent to participate in the study, followed by a demographic questionnaire. Afterwards, we attached EEG electrodes and placed participants in front of a 27 in, 1920 × 1080 px W‐LED monitor (BENQ EW2740L) with a refresh rate of 60 Hz, where the experimental task began, lasting about 60 min. Auditory stimuli were presented via dynamic stereo headphones (Sennheiser HD 201). Participants were informed prior to the experiment that they would receive 25 € or, in the case of psychology students, course credit. The money earned in the feedback learning task was thus not paid out in the end and was only virtual. The study was approved by the ethics committee of the Faculty of Mathematics and Natural Sciences at Heinrich Heine University Düsseldorf, Germany, and is in accordance with the declaration of Helsinki.

The software Presentation (Neurobehavioral Systems Inc 2020.) controlled the timing of stimulation and the recording of responses. Responses were performed on a standard computer keyboard (Logitech K120) where participants could press the left and right control keys to choose between the stimuli.

#### 2.3.1 EEG Data

EEG data was acquired from 60 active scalp electrodes, fixed with an actiCap textile softcap (BrainProducts, Germany) and evenly distributed on the scalp based on the extended 10–20 system. Electrodes were attached to the scalp sites AF3, AF4, AF7, AF8, C1, C2, C3, C4, C5, C6, CP1, CP2, CP3, CP4, CP5, CP6, CPz, Cz, F1, F2, F3, F4, F5, F6, F7, F8, FC1, FC2, FC3, FC4, FC5, FC6, FT10, FT7, FT8, FT9, Fz, O1, O2, Oz, P1, P2, P3, P4, P5, P6, P7, P8, PO10, PO3, PO4, PO7, PO8, PO9, POz, Pz, T7, T8, TP7, and TP8. The online reference was placed at the position FCz. Two further electrodes were placed over the left and right mastoids to cover as much of the scalp as possible for the calculation of the average reference (see below). Two electrodes (vEOG) were attached above (at Fp1 position) and below the left eye to measure vertical eye movements and blinks (yielding 65 electrodes in total). The ground electrode was attached to the AFz position. For data recording, a BrainAmp DC amplifier (BrainProducts, Germany) and the Brain Vision Recorder software (BrainProducts, Germany) were used, with a sampling rate of 1000 Hz and an online lowpass filter of 100 Hz. Impedances were kept below 15 kΩ.

### 2.4 Data Analysis

#### 2.4.1 Behavioral Data Analysis

The dependent variable for behavioral data analysis was response accuracy, with correct responses coded as 1 and incorrect responses as 0 for the statistical analysis (see below). Correct responses were defined as the choice of the stimulus associated with the higher reward probability. We applied generalized linear mixed‐effects models (GLME) suitable for binomial distributions and single‐trial data by means of the lme4 package (version 1.1.34; Bates et al. 2015) in R to analyze the behavioral data (The R Foundation 2021). Descriptive data visualizations were adapted with the assistance of OpenAI's GPT‐4 (OpenAI 2023). The model comprised fixed‐effect predictors of the categorical factors Stimulus Modality (visual [−0.5] vs. auditory [0.5]) and Feedback Timing (immediate [−0.5] vs. delayed [0.5]), as well as the continuous factor learning block (1 [−0.5], 2 [−0.167], 3 [0.167], 4 [0.5]) and all possible interactions between the factors. Participants were included as random intercepts. For the inclusion of random‐effect slopes per participant, we followed best practice (Meteyard and Davies 2020): all within‐subject main and interaction effects were included as random slopes, unless their inclusion led to non‐successful model fit. The best possible model was determined by using the buildmer (Version 2.11; Voeten 2020) function and resulted in the model presented in Table S1 of the Supporting Information.

#### 2.4.2 Modeling of PEs

We derived single‐trial values of the PE for each participant by fitting a reinforcement learning model to the behavioral data using MATLAB version R2021a (The MathWorks, Inc 2021; for a similar approach see Burnside et al. 2019; Lefebvre et al. 2017; Weber and Bellebaum 2024). Aiming for a model whose predicted choices deviate the least from our participants' behavior, we compared two models of different complexity. Starting point was each participants' sequence of choices and the received feedback. The PE $\delta_{c,t}$ was calculated as:

$$\delta_{c,t}=r_{t}-Q_{c,t}$$

where in a given trial t the reward $r_{t}$ is 1 for positive feedback and 0 for negative feedback, and $Q_{c,t}$ is the value of the chosen stimulus. Separately for each of the eight stimulus pairs (four containing visual and four containing auditory stimuli), both stimuli were initially assigned a stimulus value of 0.5, that was iteratively updated in every trial t in which the stimulus pair was presented. In a first model (M_1_) the stimulus value of the chosen stimulus, $Q_{c}$, was updated based on the deviation between the prior value and the received outcome, i.e., the PE δ, and a learning rate α (specific for each stimulus pair), which indicates the extent to which the PE was used to update the stimulus value.

$$Q_{c,t+1}=Q_{c,t}+\alpha *\delta_{c,t}$$

As both stimuli of a pair were always presented together, we expected participants to draw conclusions about the unchosen stimulus from feedback for the chosen stimulus. Therefore, the value of the unchosen stimulus, $Q_{u}$, equaled $1-Q_{c}$ and was updated accordingly.

For each trial, $t_{1,\ldots ,n_{\text{trials}}}$, the probability p that the model would choose the stimulus which was indeed chosen by the participant was calculated using the softmax function based on prior stimulus values of the two stimuli that were available, i.e., values of the chosen stimulus, $Q_{c,t}$, and the unchosen stimulus in trial t, $Q_{u,t}$, and an exploration parameter β:

$$p_{c,t}=\frac{e^{\mathrm{Qc},t*\beta }}{e^{\mathrm{Qc},t*\beta }+e^{\mathrm{Qu},t*\beta }}$$

with β indicating the impact of prior stimulus values on a subject's choices. A larger β indicates that a participant utilized prior stimulus values (i.e., a larger impact of prior values), whereas a smaller β indicates rather explorative choice behavior (i.e., a smaller impact of prior values).

In a next step, the probabilities *p* were used to calculate the negative summed log‐likelihood $\left(-\mathrm{LL}\right)$ as measure for the model's goodness of fit:

$$-\sum \log\left(p_{c,t_{1},\ldots ,n_{\text{trials}}}\right)$$

We used the optimization function fmincon from the Optimization Toolbox of MATLAB (R2021a, The MathWorks, Inc 2021) to minimize the −LL value by estimating values for the free parameters (α,β)/($\alpha_{\mathrm{con}},\alpha_{\mathrm{dis}},\beta$, see below) that result in the least deviation between the model's predicted choices and the participant's behavior. We fit the model repeatedly (50 iterations) to the subjects' behavior to avoid local minima. As start values for the free parameters, we allowed random numbers within the interval [0; 1]. We set value constraints for the free parameters to [0; 1] for the learning rate, and to [0; 100] for the exploration parameter β.

In a second model (M_2_), we allowed different learning rates for learning from positive feedback and negative feedback. The stimulus value of the chosen stimulus was updated with the learning rate $\alpha_{\mathrm{con}}$ for trials with positive feedback that confirms the choice as follows:

$$Q_{c,t+1}=Q_{c,t}+\alpha_{\mathrm{con}}*\delta_{c,t}$$

Analogously, for trials with negative feedback that disconfirms the choice, the stimulus value of the chosen stimulus was updated with the learning rate $\alpha_{\mathrm{dis}}$:

$$Q_{c,t+1}=Q_{c,t}+\alpha_{\mathrm{dis}}*\delta_{c,t}$$

Everything else stayed the same compared to M_1_.

The two models were compared based on their negative summed log‐likelihood $\left(-\mathrm{LL}\right)$ by a paired samples *t*‐test. M_2_ resulted in significantly lower −LL values (M = 360.88, SD = 246.73) than M_1_ (M = 381.21, SD = 242.18), *t*(34) = 9.18, *p* < 0.001, indicating a better model fit. Furthermore, a lower Bayesian Information Criterion (BIC) indicated that M_2_ (BIC = 751.73) provides a better balance between model fit and complexity compared to M_1_ (BIC = 782.40). Eventually, M_2_ was used to extract stimulus values and trial‐by‐trial PEs. Single‐subject −LL values are illustrated in Figure S1A of the Supporting Information. Furthermore, we visualized the learning rates for positive and negative feedback ($\alpha_{\mathrm{con}}$ and $\alpha_{\mathrm{dis}}$) to ensure that they do not systematically converge to values of 0 or 1 (see Figures S1B and S2 of the Supporting Information). Finally, we examined participants' win‐stay and lose‐shift behavior to determine whether participants were using the PE to adapt their behavior. The results are presented in the Supporting Information, in the section titled Win‐stay vs. lose‐shift analysis accompanied by Figure S3. All visualizations and analyses supported that the PE modeling resulted in meaningful data.

#### 2.4.3 EEG Data Analysis

BrainVision Analyzer 2.2 (Brain Products GmbH 2018), MATLAB R2021a (The MathWorks, Inc 2021) and R (The R Foundation 2021) were used for EEG data analysis. Trials in which participants failed to answer (M = 1.67%, SD = 2.59%, Min = 0.16%, Max = 13.44%) were excluded from any further EEG analyses.

##### 2.4.3.1 Preprocessing

In a first step, we re‐referenced the data to the average of all 63 scalp electrodes including the mastoids (see above; the signal at the online reference site FCz was calculated; see Arbel et al. 2017; Höltje and Mecklinger 2020, for similar procedures). The reduction of ERP effects that can result as a consequence of using an average reference (see Luck 2014) is minimized for high‐density EEG acquisition as in our study. In a second step, the data were filtered, using a 30 Hz low cut‐off and a 0.1 Hz high cut‐off filter (as proposed by Luck 2014) as well as a 50 Hz Notch Filter. In order to correct for blink artifacts, an independent component analysis (ICA) and reverse ICA was performed on single‐subject EEG data (see Peterburs et al. 2016; Weismüller et al. 2019 for a similar procedure). We created segments from 200 ms before to 800 ms after feedback onset and performed a baseline correction relative to the first 200 ms. Then, segments with artifacts in electrodes used to measure the FRN and N170 (see below) were removed (for a similar approach see Albrecht et al. 2023; all segments containing voltage steps > 50 μV/ms, differences between values > 80 μV or < 0.1 μV within an interval of 100 ms or amplitudes > 80 μV or < −80 μV; M = 1.09%, SD = 2.23%, Min = 0.00%, Max = 12.97%). This way, we aimed to include as much data as possible for our single‐trial analysis, as linear mixed‐effects (LME) models that we applied for the analyses (see below) are tailored for managing data variability (Bates et al. 2015; Quené and Van den Bergh 2004). On average, per participant, 156.6 trials (SD = 5.2, Min = 130, Max = 160) from the visual task with immediate feedback and 155.6 trials (SD = 5.3, Min = 136, Max = 160) with delayed feedback entered the analysis. From the auditory task, on average 155.6 trials (SD = 7.6, Min = 122, Max = 160) with immediate feedback and 154.2 trials (SD = 8.5, Min = 112, Max = 160) with delayed feedback entered the analysis per participant.

The remaining segments were grouped and averaged for each of the eight conditions (positive and negative immediate feedback and delayed feedback for the tasks involving visual and auditory stimuli), yielding eight averages per participant. Subsequently, all single‐trial segment data as well as all averages per condition and participant were exported for later analysis. For further preprocessing steps, MATLAB scripts (MathWorks, MA) were utilized, which were adapted with the assistance of OpenAI's GPT‐4 (OpenAI 2023) to extract single‐trial data.

For the N170, single‐trial amplitudes (see Albrecht et al. 2023) were derived from electrodes P7 and P8 (see Arbel et al. 2017; Höltje and Mecklinger 2020; Kim and Arbel 2019), as outlined in the preregistration for the study (osf.io/fu2gy). First, the maximum negative peak amplitude between 130 and 230 ms post‐feedback was determined in each participant's average, at both electrode sites and for all eight conditions separately (see above). Then, for each single trial, the mean amplitude in a time window of ±10 ms around the condition‐specific N170 peak latency was calculated. Because grand averages revealed differences between the conditions already in the preceding positive peak (see Figure 3), we additionally extracted the single‐trial mean amplitude in a time window of ±10 ms around the preceding positive peak (P1). As for the negative peak, the latency of the P1 was determined based on the condition‐specific average at each electrode site. The P1 was determined as the maximum positivity in a time window starting 80 ms after feedback onset to the respective condition‐specific negative peak. For the analysis, we used the N170 defined as the peak‐to‐peak amplitude by subtracting the single‐trial amplitude value of the preceding P1 from the single‐trial value of the negative peak.

For the FRN, single‐trial amplitudes were derived from an electrode cluster consisting of Fz, FCz, Cz, FC1, and FC2, for which the signal was pooled. Previous studies showed that the FRN was maximal at FCz but also pronounced at neighboring channels (Arbel et al. 2017; Kim and Arbel 2019; Maurer et al. 2022; Mushtaq et al. 2022). To account for individual differences, we decided to measure FRN amplitudes in the pooled signal of a group of five frontocentral electrode sites (for a similar approach see Zottoli and Grose‐Fifer 2012), including FCz and neighboring electrodes (see Weber and Bellebaum 2024). For each participant, we used their mean waveform for both positive and negative feedback separately for each of the four conditions (immediate feedback in the visual task, delayed feedback in the visual task, immediate feedback in the auditory task, and delayed feedback in the auditory task). Then, we computed the difference wave by subtracting the mean positive feedback waveform from the mean negative feedback waveform for each of these four conditions. For each participant, we identified the maximum negative peak amplitude in each of the four difference waves within a time window of 230–360 ms post‐feedback, i.e., the peak latency was determined separately for each condition. Next, for each single trial, we extracted the mean amplitude within a ±10 ms window around the condition‐specific difference wave peak latency. It is important to emphasize that our dependent variable is not derived from the difference wave itself. Rather, the difference wave was only used to identify the latency at which the difference between the processing of positive and negative feedback is maximal. This latency was then used to extract the single‐trial ERP data. Therefore, our actual dependent variable was derived from the ERPs for positive and negative feedback in each condition.

##### 2.4.3.2 Statistical Analysis

###### 2.4.3.2.1 N170

The single‐trial N170 amplitude was analyzed as a dependent variable by applying an LME analysis in R (Bates et al. 2015). The model comprised fixed‐effect predictors of the categorical factors feedback timing (immediate [−0.5] vs. delayed [0.5]), stimulus modality (visual [−0.5] vs. auditory [0.5]) and feedback valence (negative [−0.5] vs. positive [0.5]). Furthermore, the PE was used as a continuous predictor. However, as the signed PE is confounded by valence, we used the unsigned or absolute PE (scaled and mean centered, yielding negative values for PE values below the mean vs. positive values for PE values above the mean) indicating general expectation violations or surprise. Finally, the factor electrode (P7 [−0.5] vs. P8 [0.5]) was added. Furthermore, we added all possible interactions between the factors. Although this adds complexity to the model, we believe that this is justified due to our hypotheses and the interrelated nature of the predictors. Our hypothesis concerning the N170 already involves an interaction between the factors stimulus modality, feedback timing, and electrode, as we expected its amplitude to be most pronounced for delayed feedback following choices between visual stimuli and over the right hemisphere. The more exploratory analysis, whether the N170 encodes a PE, aims at the question of whether there is an interaction between feedback valence and the absolute PE. Moreover, this interaction may again be modulated by the three factors Stimulus Modality, feedback timing, and electrode. Given that all of the predictors are thus closely linked and may influence each other, we decided to include all interactions when planning the study (as was also preregistered). Participant was included as a random‐effect factor. Random slopes per participant were added as described for the behavioral GLME above (see Table S1 of the Supporting Information for the resulting model). Simple slope analyses were performed to resolve significant interactions, with Bonferroni‐corrected *p*‐values (multiplied by the number of conducted tests).

###### 2.4.3.2.2 FRN

The single‐trial FRN amplitude was analyzed as a dependent variable by applying an LME analysis in R (Bates et al. 2015). The model comprised fixed‐effect predictors of the categorical factors feedback timing (immediate [−0.5] vs. delayed [0.5]), stimulus modality (visual [−0.5] vs. auditory [0.5]) and feedback valence (negative [−0.5] vs. positive [0.5]) and as a continuous factor the mean centered unsigned PE, as well as all possible interactions between the factors. For the FRN, it has been shown that its amplitude reflects a (signed) PE, indicated by the interaction between the factors feedback valence and (unsigned) PE. Moreover, effects of feedback timing have been found, which may also interact with PE coding (Weber and Bellebaum 2024). In this study, we aimed to explore whether stimulus modality affects the FRN, alone or in interaction with the mentioned factors. Participant was included as a random‐effect factor. Random slopes per participant were added as described for the behavioral GLME above (see Table S1 of the Supporting Information for the resulting model). Significant interactions were resolved as described for the N170 (see above).

## 3 Results

### 3.1 Behavioral Results

With the GLME analysis of the behavioral data, we first aimed to determine whether participants learned to increasingly select the more frequently rewarded stimulus across the four learning blocks. Second, we examined whether there were any differences in learning between the tasks involving choices between visual and auditory stimuli, or between the conditions with immediate and delayed feedback, or between any combinations of these two factors.

**FIGURE 2:**
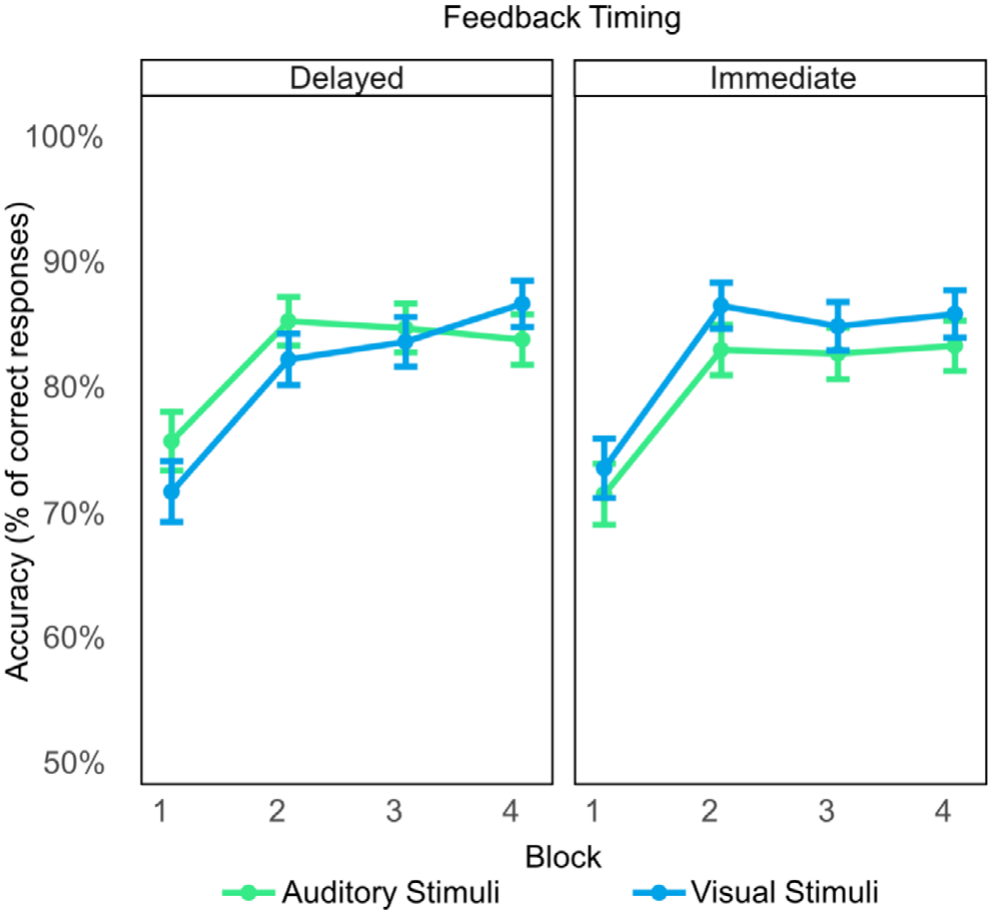
Descriptive pattern of performance improvement during the feedback learning task. Mean accuracy (% of correct responses) for the four learning blocks of the probabilistic feedback learning task, separately for immediate and delayed feedback and for the tasks involving choices between visual and auditory stimuli. Error bars represent 95% confidence intervals.

Descriptive data are presented in Figure 2. Table S2 in the Supporting Information lists β‐estimates and effect‐specific *z*‐tests for the GLME analysis investigating effects of feedback timing, feedback valence, and stimulus modality on the behavioral data. The analysis revealed a significant effect of Block (*p* < 0.001) on response accuracy, driven by an increasing number of correct responses across the four learning Blocks. Figure 2 suggests that this effect is due in particular to an increase in correct responses from block 1 to block 2. No other significant effects were observed (all *ps* ≥ 0.140), indicating that learning was comparable for immediate and delayed feedback and for the tasks involving choices between visual and auditory stimuli.

### 3.2 EEG Results

#### 3.2.1 N170

**FIGURE 3:**
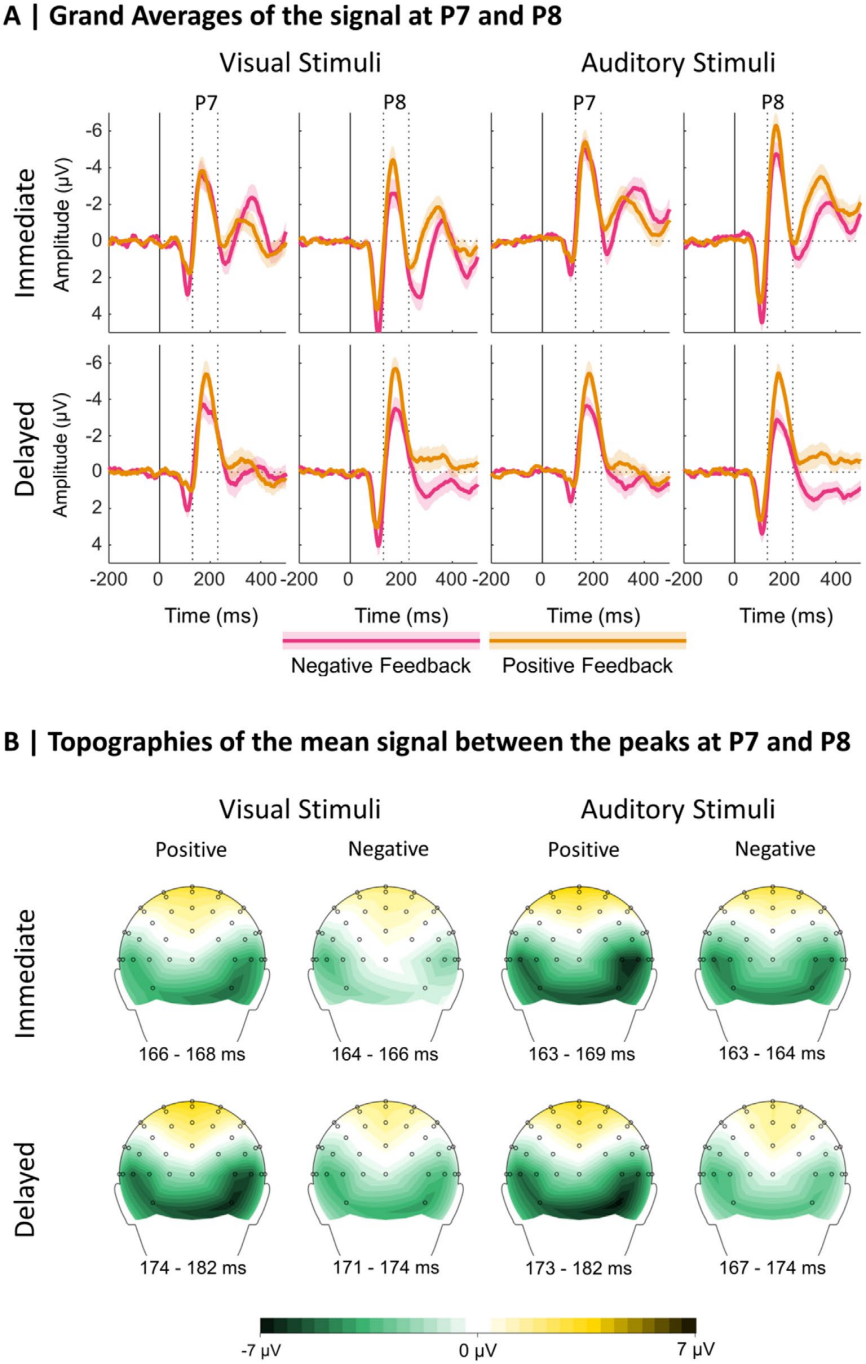
Grand averages at P7 and P8 and topographical maps at the respective peaks. (A) Grand Averages: Dotted lines indicate the time window used for the N170 peak detection. Shaded areas indicate standard errors. (B) Topographies: The maps are based on the condition‐specific N170 peaks.

With the LME analysis of the N170 single‐trial data, we aimed to test our hypothesis that the N170 is most pronounced for delayed feedback referring to the choice of visual stimuli, with a possibly more pronounced effect over the right hemisphere. This would be reflected in an interaction between the factors stimulus modality, feedback timing, and electrode. Moreover, we aimed to investigate if the N170 reflects a signed PE, which would be reflected in an interaction between feedback valence and the unsigned PE, and whether this effect is modulated by the other factors stimulus modality, feedback timing, and electrode. The analyses thus focused on interaction effects of the involved predictors, and main effects will not be reported in the following. Grand averages for the ERPs following positive and negative immediate and delayed feedback for the choice between visual and auditory stimuli at electrode sites P7 and P8 are presented in Figure 3. In addition, the Supporting Information contains grand averages separately for low and high absolute PE values (expected vs. unexpected; Figure S4). Table S3 in the Supporting Information lists β‐estimates and effect‐specific *t*‐tests for all effects of the LME analysis investigating the N170 amplitude. In the following, more negative N170 amplitudes are described as more pronounced or larger, respectively.

**FIGURE 4:**
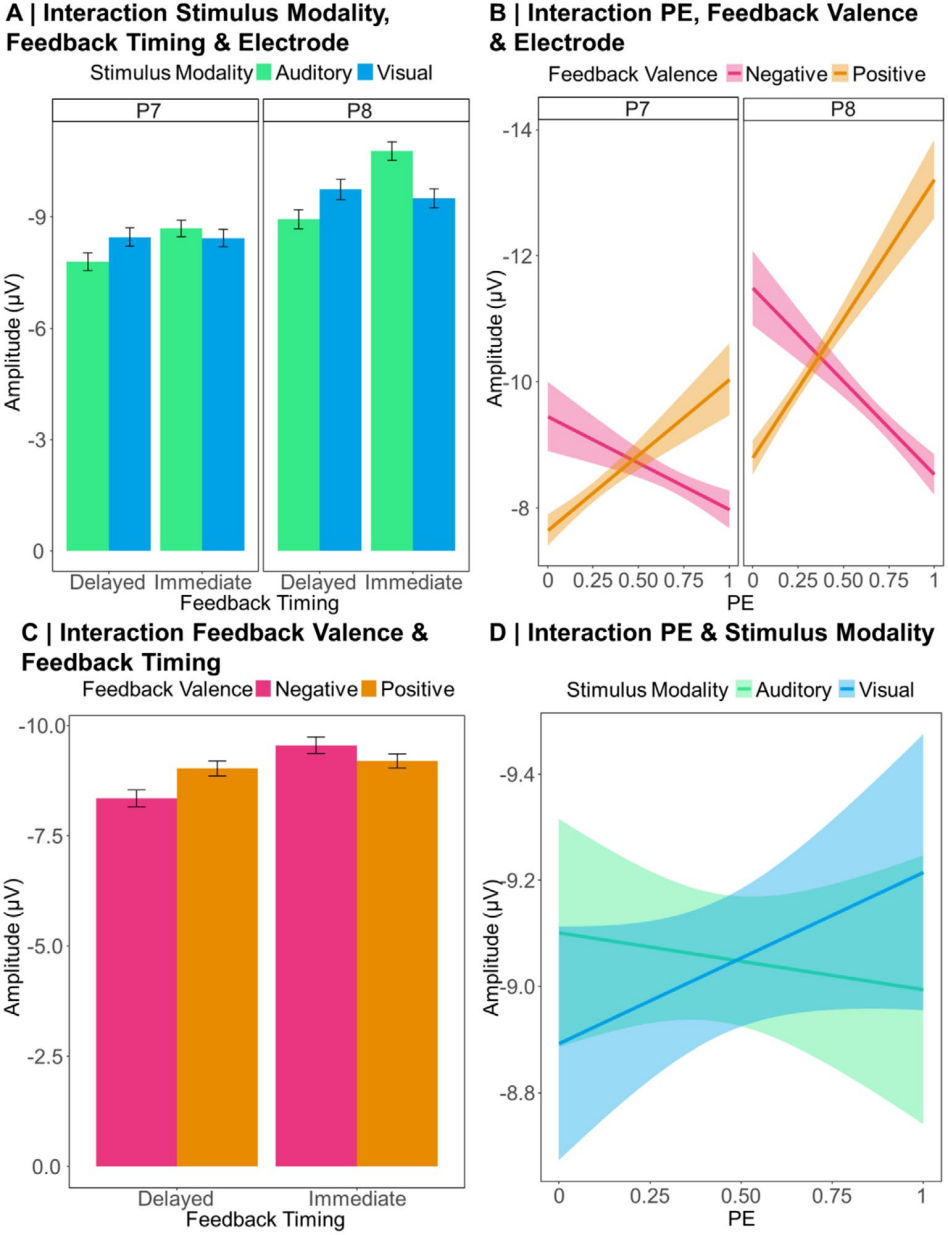
Descriptive data patterns underlying the N170 analysis. Error bars indicate a 95% confidence interval. Shaded areas indicate standard errors.

Regarding our hypothesis, we indeed found a significant stimulus modality × feedback timing interaction (*p* < 0.001) that was further explained by a significant stimulus modality × feedback timing × electrode interaction (*p* < 0.001) which we thus resolved. The descriptive pattern behind the three‐way interaction is presented in Figure 4A. A simple slope analysis revealed that for the P7, the effect of Stimulus Modality was neither significant for immediate (β = −0.08, SE = 0.33, *t* = −0.23, *p* > 0.999) nor for delayed feedback (β = 0.72, SE = 0.42, *t* = 1.72, *p* = 0.363). For the P8, there was a significant effect of stimulus modality following immediate feedback with larger N170 amplitudes for auditory compared to visual stimuli, β = −1.29, SE = 0.33, *t* = −3.93, *p* < 0.001. For delayed feedback, this effect was reversed with significantly larger N170 amplitudes for visual compared to auditory stimuli, β = 1.31, SE = 0.42, *t* = 3.14, *p* = 0.010.

With regard to more exploratory results, we found a significant interaction between PE and feedback valence (*p* < 0.001), indicating that the N170 indeed reflects the signed PE. As this interaction was further explained by a significant three‐way interaction between PE, feedback valence, and electrode (*p* = 0.001), we decided to resolve the three‐way interaction with simple slope analyses. The underlying descriptive data are presented in Figure 4B. For P7, the PE had no significant effect on the N170, neither for negative (β = 0.18, SE = 0.40, *t* = 0.46, *p* > 0.999) nor for positive feedback (β = −0.66, SE = 0.41, *t* = −1.60, *p* = 0.439). For P8, the PE had a significant effect on the N170 following negative feedback, with larger amplitudes for expected compared to unexpected feedback (β = 1.23, SE = 0.40, *t* = 3.11, *p* = 0.008). For positive feedback, the effect was reversed, with significantly larger N170 amplitudes for unexpected compared to expected feedback (β = −2.27, SE = 0.41, *t* = −5.52, *p* < 0.001).

**FIGURE 5:**
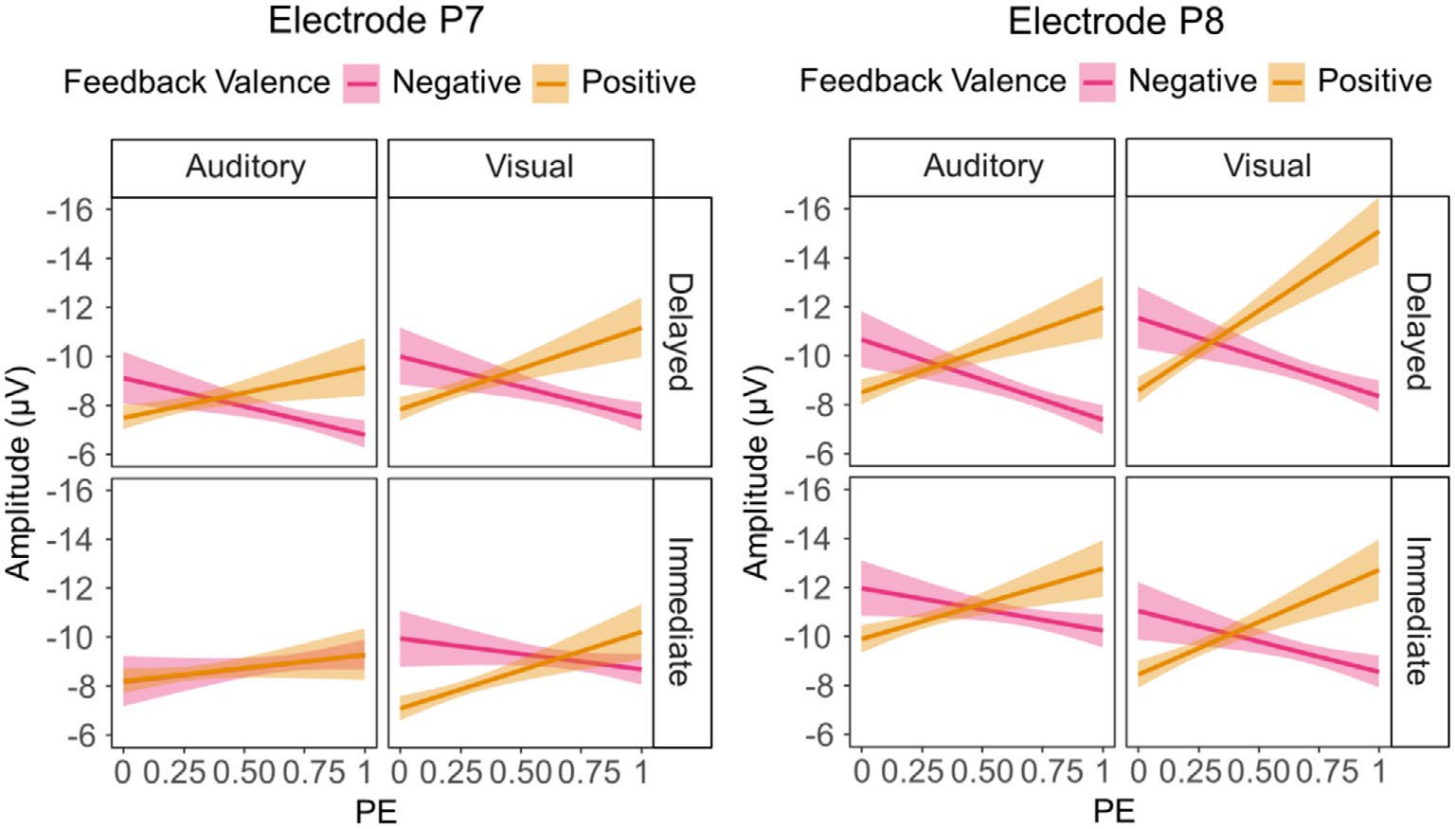
Descriptive data pattern underlying the PE × Feedback Valence × Modality × Feedback Timing × Electrode interaction for the N170. Shaded areas indicate standard errors.

While we found two further two‐way interactions, one between feedback valence and feedback timing (*p* = 0.002, the underlying descriptive data are presented in Figure 4C) and one between PE and stimulus modality (*p* = 0.026, the underlying descriptive data are presented in Figure 4D), a significant five‐way interaction between all included predictors was of main interest (*p* = 0.042). The underlying descriptive pattern is presented in Figure 5.

To resolve this interaction, we split the dataset based on the electrode and repeated the LME analysis separately for P7 and P8. There was a significant four‐way interaction between the remaining factors feedback timing, feedback valence, stimulus modality, and PE for the P8 (β = 4.88, SE = 2.36, *t*(2251.33) = 2.07, *p* = 0.039), but not for the P7 (β = −2.68, SE = 2.18, *t*(2767.86) = −1.23, *p* = 0.218). To resolve the four‐way interaction at P8, we again split the dataset, but this time according to Feedback Timing. For delayed feedback, the three‐way interaction between feedback valence, stimulus modality, and PE reached significance (β = 5.07, SE = 1.76, *t*(7454.36) = 2.87, *p* = 0.004), unlike for immediate feedback (β = 0.98, SE = 1.67, *t*(5272.01) = −0.58, *p* = 0.560). To resolve the three‐way interaction for delayed feedback, we finally split the dataset according to stimulus modality. We found a significant interaction between PE and feedback valence for visual stimuli (β = −7.12, SE = 1.30, *t*(4870.20) = −5.45, *p* < 0.001), but not for auditory stimuli (β = −1.36, SE = 1.23, *t*(4300.97) = −1.11, *p* = 0.267). We resolved the two‐way interaction found for visual stimuli with a simple slope analysis. For negative feedback, larger PE values led to significantly less pronounced N170 amplitudes (β = 2.02, SE = 0.87, *t* = 2.32, *p* = 0.046). For positive feedback, larger PE values led to significantly more pronounced N170 amplitudes (β = −5.09, SE = 0.93, *t* = −5.47, *p* < 0.001). To conclude, the five‐way interaction is driven by a reflection of the PE in the N170 measured over the right hemisphere, especially following delayed feedback that refers to visual stimuli. In the Supporting Information, the descriptive data underlying the N170 interaction effects described in the main text are represented with a detailed overview of data distribution and variance (see Figures S5 and S6).

#### 3.2.2 FRN

**FIGURE 6:**
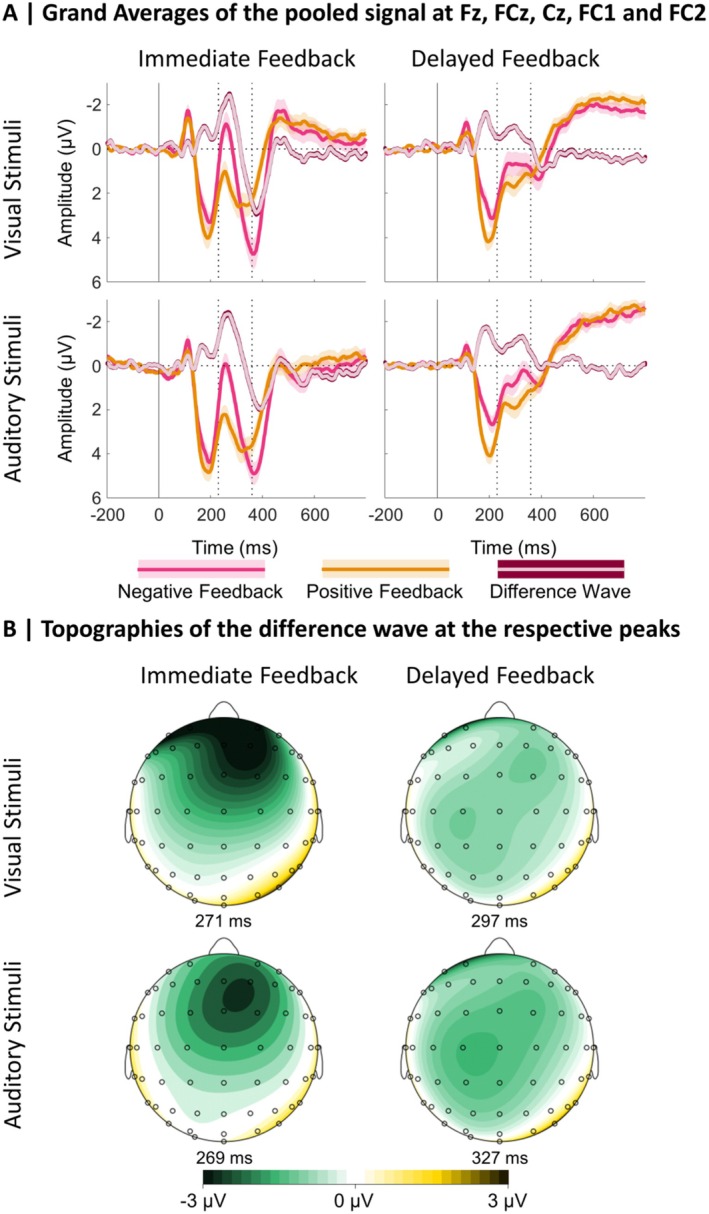
Grand averages and topographical maps of the FRN. (A) Grand Averages: Dotted lines indicate the time window used for the peak detection in the difference wave (negative—positive feedback). Shaded areas indicate standard errors. (B) Topographies: The maps are based on the condition‐specific difference wave.

**FIGURE 7:**
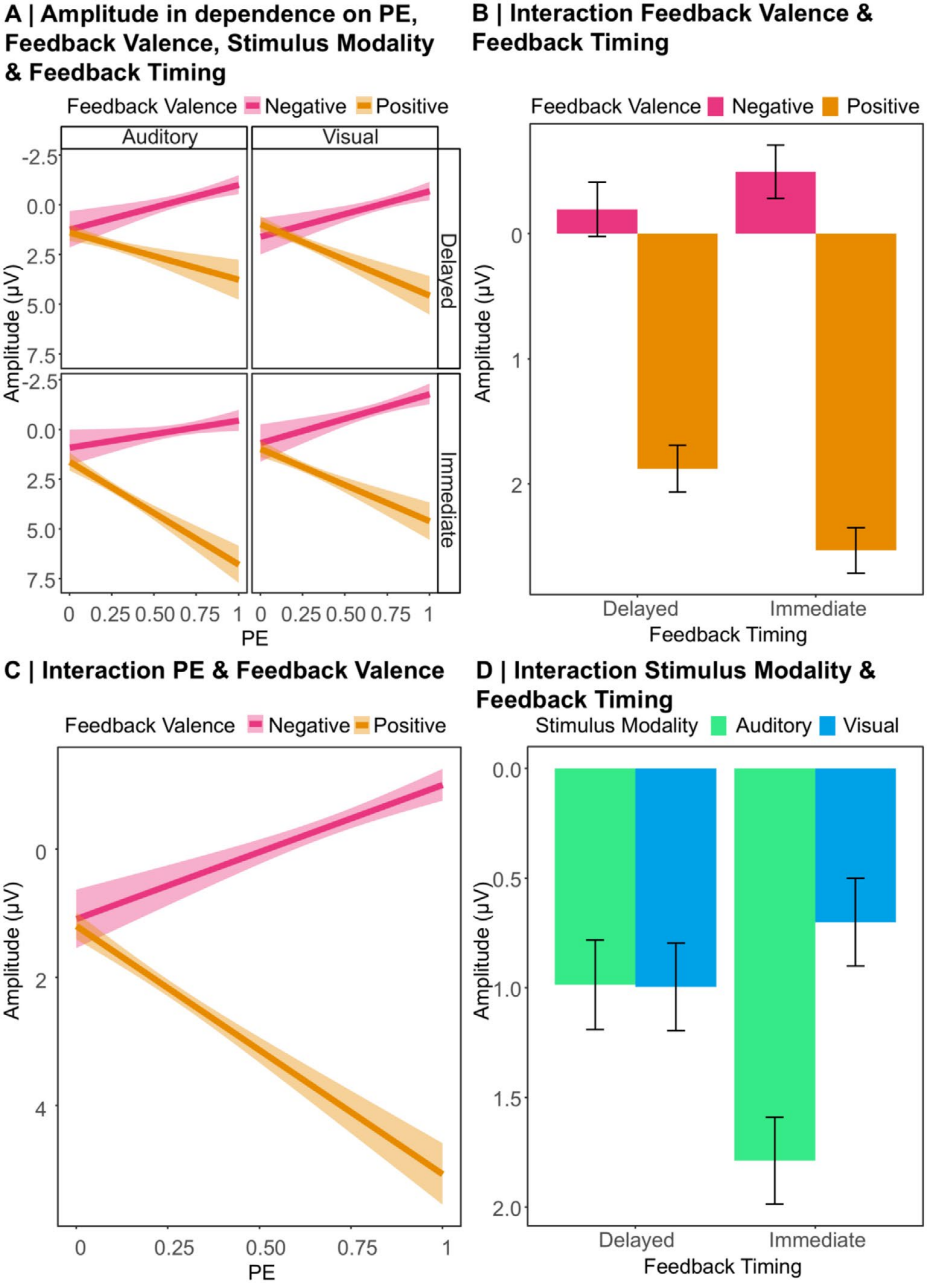
Descriptive data patterns underlying the FRN analysis. Error bars indicate a 95% confidence interval. Shaded areas indicate standard errors.

With the LME analysis of the FRN single‐trial data, we aimed to replicate that the FRN is sensitive to feedback valence, especially following immediate feedback. Furthermore, we aimed to replicate that the amplitude reflects a signed PE signal, reflected in an interaction between (unsigned) PE and feedback valence. In an exploratory manner, we were also interested in the effects of stimulus modality, alone or in interaction with the other predictors. Grand averages for the ERPs following positive and negative immediate and delayed feedback for the choice between visual and auditory stimuli pooled over the frontocentral cluster of electrodes are presented in Figure 6. In addition, the Supporting Information contains grand averages separately for expected and unexpected feedback (Figure S7). For β‐estimates of the LME analysis on the FRN amplitude and effect‐specific *t*‐tests, see Table S4 in the Supporting Information. Descriptive statistics can be found in Figure 7A. In the following, more negative FRN amplitudes are described as more pronounced or larger, respectively.

As expected, we could replicate previous findings of a significant main effect of feedback valence (more pronounced FRN for negative compared to positive feedback, *p* < 0.001) and a significant feedback timing × feedback valence interaction (*p* = 0.006, see Figure 7B). Resolving this interaction using simple slope analyses showed a more pronounced FRN for negative compared to positive feedback for both immediate (β = 2.94, SE = 0.39, *t* = 7.60, *p* < 0.001) and delayed feedback (β = 1.86, SE = 0.26, *t* = 7.16, *p* < 0.001), with a larger feedback valence effect for immediate feedback.

Another replication concerned a significant feedback valence × PE interaction (*p* < 0.001, see Figure 7C). Resolving this interaction via simple slope analyses resulted in a significant effect of the PE for negative feedback (β = −3.55, SE = 0.63, *t* = −5.60, *p* < 0.001) with larger (i.e., more negative) FRN amplitudes for unexpected feedback. For positive feedback, there was a significant effect of the PE with smaller (i.e., more positive) amplitudes for unexpected feedback (β = 3.89, SE = 0.58, *t* = 6.73, *p* < 0.001).

Regarding more exploratory results involving the factor stimulus modality, the analysis revealed a significant main effect (*p* = 0.014), which was further explained by a significant feedback timing × stimulus modality interaction (*p* = 0.002, see Figure 7D). We resolved this interaction using a simple slope analysis that yielded a significant effect of Stimulus Modality, with larger amplitudes for visual than auditory stimuli, for immediate (β = 1.33, SE = 0.31, *t* = 4.33, *p* < 0.001), but not for delayed feedback (β = −0.04, SE = 0.34, *t* = −0.13, *p* > 0.999). All other main and interaction effects were not significant (all *ps* ≥ 0.171). In the Supporting Information, the descriptive data underlying the FRN interaction effects described in the main text are represented with a detailed overview of data distribution and variance (see Figure S8).

## 4 Discussion

The present study aimed to investigate whether the N170 ERP component is modulated by the modality of the associated stimulus during feedback processing in a reinforcement learning task. While previous studies have examined the influence of feedback modality (visual vs. auditory, see Kim and Arbel 2019), our study is the first to manipulate the sensory modality of the stimuli (visual vs. auditory) between which participants make their choices before receiving visual feedback. More specifically, we hypothesized that the N170 reflects a process that bridges the temporal gap between the choice of a stimulus and feedback, especially for delayed feedback for visual stimuli that are associated with the feedback and over the right hemisphere. Indeed, we found that delayed feedback related to the choice of visual stimuli led to significantly larger N170 amplitudes than feedback following the choice of auditory stimuli over the right lateral hemisphere. Furthermore, we found pronounced effects of the PE on the N170 measured over the right hemisphere, again especially for delayed feedback related to the choice of visual stimuli. For immediate feedback, however, an unexpected pattern emerged, with larger N170 amplitudes for feedback following the choice between auditory compared to visual stimuli. Regarding the FRN, we also found a modality effect, specifically for immediate feedback: it was more pronounced when the feedback was related to the choice between visual stimuli than auditory stimuli. Despite the differences in feedback processing depending on Feedback Timing and Stimulus Modality, our participants appeared to learn equally well from immediate and delayed feedback, as well as in the tasks involving visual or auditory stimuli.

### 4.1 The Role of the Modality of the Associated Stimulus for Feedback Processing

Based on previous studies, a clear functional interpretation of the N170 in the context of (delayed) feedback processing is not yet possible. In studies investigating delayed feedback processing, the stimuli associated with feedback were always visual (Arbel et al. 2017; Höltje and Mecklinger 2020; Kim and Arbel 2019). We hypothesized that the modality of the stimulus that is associated with the feedback modulates the amplitude of the N170. Since this component has been linked to visual processing in the extrastriate cortex (Brem et al. 2006; Deffke et al. 2007; Gao et al. 2019; Iidaka et al. 2006), we assumed that the N170 reflects a reactivation of a visual stimulus associated with feedback and should be more pronounced when feedback is given for a choice between visual stimuli, especially when feedback is delayed. Given that the right hemisphere plays a dominant role in processing certain visual stimuli, such as faces (Rossion 2014), and in N170 generation in different contexts (Baker and Holroyd 2009, 2013; Baker et al. 2015; Kim and Arbel 2019), we were particularly interested in whether the effects would be stronger over the right hemisphere.

The research question of the present study thus addresses the implementation of the so‐called credit assignment problem within the brain. For immediate feedback, the temporal proximity of the reward signal from the dopaminergic midbrain and the activation of cortical areas representing, e.g., a visual stimulus probably suffice to establish a connection. (Schultz 2002; Jocham et al. 2016) found heuristic time‐based learning mechanisms related to activity in circuits including the striatum. Furthermore, reward signals coded by dopamine drive synaptic connections—the molecular basis of learning—in the striatum in a narrow time window of up to 2 s (Yagishita et al. 2014). However, if feedback is presented after a longer delay, the representation of the selected stimulus might be reactivated at the time of feedback presentation. The present study provides first evidence that the modality of the associated stimulus affects the N170: In the right hemisphere, we found larger N170 amplitudes following delayed feedback for the choice of visual compared to auditory stimuli. While a study by Herholz et al. (2012) found an overlap of melody perception and imagery in secondary auditory areas, supporting the existence of auditory reactivation processes, the N170 has been specifically linked to stimulus processing in the visual domain (Bentin et al. 1996; Itier and Taylor 2004; Kloth et al. 2013; for reviews see Yovel 2016; Carreiras et al. 2014). Our results thus support the hypothesis that the N170 reflects stimulus reactivations in higher‐order visual areas, which may mirror an association mechanism in which reactivated representations of a selected stimulus are used to bridge the temporal gap to delayed feedback. This interpretation is in line with fMRI studies that revealed post‐reward reactivation mechanisms in visual (Schiffer et al. 2014) as well as somatosensory areas (Pleger et al. 2008, 2009) as a way to assign credit to a stimulus for an obtained reward. Finding this potential reactivation for the N170 only over the right hemisphere may be due to the functional specialization of the right hemisphere for visuo‐spatial processing (e.g., Thiebaut de Schotten et al. 2011), as the visual stimuli used in our study (hiragana characters) had a visuo‐spatial character. Furthermore, studies investigating the N170 in the context of navigational feedback learning particularly linked it to activity within the right MTL, or more precisely the right parahippocampal cortex (Baker and Holroyd 2009, 2013; Baker et al. 2015). It is important to note that the functional meaning of the N170 could be different in contextually different tasks.

Against our expectation, we found a larger feedback‐locked N170 for choices between auditory than visual stimuli for immediate feedback. One explanation could be that the N170 reflects overlapping activity of MTL and extrastriate visual areas in feedback processing. Indeed, the hippocampus has been found to be involved in feedback processing even for short feedback delays of only two seconds (Dickerson et al. 2011). Integrating information about feedback and the associated stimulus, hippocampal processing demands for the auditory condition may have been particularly high, as this condition required cross‐modal associations, which activates the hippocampus more than unimodal associations (Butler and James 2011). For delayed feedback, the extrastriate visual cortex contribution to the N170 may have been higher.

For the FRN, which has been investigated much more extensively in the context of feedback processing, the fact that we found larger FRN amplitudes following immediate feedback for the choice between visual compared to auditory stimuli was also surprising. FRN effects are mainly interpreted with respect to feedback valence and/or the PE. As stimulus modality did not affect the effects of feedback valence or the reflection of the PE in the FRN, it is questionable whether stimulus modality exerted a significant influence on the processes underlying the FRN.

### 4.2 Effects of Feedback Valence and PE for Immediate and Delayed Feedback

In contrast to previous studies, we did not find a main effect of feedback timing (Arbel et al. 2017; Kim and Arbel 2019; Höltje and Mecklinger 2020) or feedback valence (Kim and Arbel 2019) for the N170, but an interaction between the two: a valence effect was only detectable when feedback was delayed. In this regard, the N170 formed a kind of counterpart to the FRN, for which there was an enhanced differentiation between immediate positive and negative feedback compared to delayed (for similar results see Arbel et al. 2017; Höltje and Mecklinger 2020; Peterburs et al. 2016; Weinberg et al. 2012; Weismüller and Bellebaum 2016).

This complementary processing is further evident considering the PE effects on the two components. Effects of reward PEs on the N170 have not been reported before. We found that the N170 reflects the whole range of PEs, which is in line with recent findings by Baker et al. (2021, 2023), who reported more pronounced N170 amplitudes for unpredictable compared to predictable stimuli during the perceptual processing of visual stimuli, linking the N170 to surprise in general. While the FRN also reflected the whole range of PEs in the present study, the N170, especially over the right hemisphere, was enhanced for unexpected positive feedback and reduced for unexpected negative feedback, and the pattern of PE coding was reversed for the FRN that became more negative when negative feedback was unexpected and more positive when positive feedback was unexpected. Regarding the N170, enhanced amplitudes following unexpected positive feedback might indicate that representations of unexpectedly rewarded stimuli are especially reactivated. Put simply, this means that it is especially important to remember which stimulus brought the reward and strengthen that relationship. Remembering what led to the reward can be very helpful for survival, and a form of reactivation following rewards could be a way to bind them to preceding situations (Singer and Frank 2009).

Correlates of the PE in the N170 could be interpreted as reflecting PE‐related hippocampal activity (Dickerson et al. 2011; Foerde and Shohamy 2011). The midbrain dopamine system contains neurons that have widespread projections and could send reinforcement signals not only to the striatum and frontal cortex (Schultz 2002) but also to the hippocampus (Schott et al. 2004). Zaghloul et al. (2009) observed that the firing rate of neurons in the human substantia nigra was higher for unexpected gains compared to losses as early as 150 ms after feedback presentation. This finding supports the possibility that the PE effects observed in the N170, which had a latency of about 160 ms to 180 ms in the present study, could reflect the influence of the dopaminergic midbrain on the MTL, specifically in the context of feedback‐based learning. However, alternative explanations are also possible. For instance, the locus coeruleus (LC), which plays a key role in norepinephrine release, also reacts to unexpected events that evoke attention like rewards, sending PE signals to other areas of the brain, for example via axons diverging to the cerebral cortex (for a review see Schultz and Dickinson 2000). Importantly, the LC also projects to the hippocampus, where its norepinephrine projections have been shown to modulate synaptic plasticity, playing a crucial role in regulating behavioral control (for reviews, see Sara 2009, 2015; Schultz and Dickinson 2000).

Nevertheless, finding a pronounced PE effect on the N170 for the prior choice between visual and not auditory stimuli and especially for delayed feedback supports the role of the N170 in the processing of visual stimuli and the idea that it specifically represents a reactivation of visual areas during feedback processing. Since signals from the MTL may evoke the reactivation of an internal representation of an event, allowing it to be linked to a later event such as the feedback in our task (Qin et al. 2007), we propose that the N170 reflects overlapping activity of the MTL and extrastriate visual areas.

For the signal in the FRN time window, accumulating evidence suggests that it is specifically modulated by positive feedback. Early studies showed that the ERP response to losses and breaking even (neither winning nor losing) can be understood as the baseline response, while rewards evoke a relative positivity (Holroyd et al. 2006; Kujawa et al. 2013). This suggests the unfolding of a positivity on gain trials more than a negativity during loss trials, in accordance with the conception of the RewP (Proudfit 2015). In line with this, it was reported that the PE affected positive feedback, while no effect emerged for negative feedback (Weber and Bellebaum 2024; Kirsch et al. 2022). In the present study, however, the signal in the FRN/RewP time range also reflected the full range of PEs, irrespective of feedback delay. The differential contribution of PE signals reflected in the FRN/RewP and the N170, and thus of the activity in neural structures underlying these components, to learning remains to be explored in future studies.

### 4.3 Limitations

One aspect that limits the generalizability of our results is our predominantly female sample. A previous study found, for example, increased punishment sensitivity for women that might lead to sex differences in negative feedback processing also in our study (Santesso et al. 2011). However, the main interest in our study was in how far the modality of the feedback‐preceding stimulus affects feedback processing in interaction with feedback timing, and we have no reason to believe that the effects related to this research question are affected by sex. Nevertheless, potential sex differences could be investigated in future studies.

Another concern is that the reported valence effects may partly be driven by perceptual differences between positive and negative feedback. The feedback color was not counterbalanced across participants, and this difference in saliency may have affected the FRN (Liu et al. 2014; Pfabigan et al. 2015) or, even more likely, the N170, which is associated with visual processing. However, the focus in our study was on interaction effects, which can hardly be caused by perceptual differences between negative and positive feedback. To rule out confounds of visual processing, future studies could consider using abstract feedback stimuli that are not inherently associated with valence, as implemented by Höltje and Mecklinger (2020), who used indoor vs. outdoor pictures to signal positive and negative feedback.

### 4.4 Conclusions

The fact that we can use feedback to adapt our behavior, even if presented after a temporal delay, is crucial for learning and progression in our complex world. A more pronounced N170 following delayed feedback related to the choice of visual compared to auditory stimuli over the right hemisphere, combined with a representation of the PE after delayed feedback for choices of visual stimuli, supports our assumption that this component reflects modality‐specific activity within higher‐order visual areas of the brain. The reactivation of the chosen stimulus' representation in visual areas, possibly initiated by regions within the MTL, could be a mechanism to establish an association between the selection of a stimulus and the temporally delayed feedback.

## Author Contributions

**Madita Röhlinger:** conceptualization, data curation, formal analysis, investigation, methodology, project administration, visualization, writing – original draft, writing – review and editing. **Christine Albrecht:** data curation, methodology, supervision, validation, writing – review and editing. **Christian Bellebaum:** conceptualization, funding acquisition, project administration, resources, supervision, validation, writing – review and editing.

## Conflicts of Interest

The authors declare no conflicts of interest.

## Supporting information


Data S1.

## Data Availability

All data supporting the findings are openly accessible through the Open Science Framework at https://osf.io/brpy7/?view_only=117f83d7d4b44448aa6aa8d7e02913d2.
