# Supplementary material for: The Role of the N170 in Linking Stimuli to Feedback—Effects of Stimulus Modality and Feedback Delay
Source: Psychophysiology. 2025 Apr 15;62(4):e70050. doi: 10.1111/psyp.70050 (PMC11998638; doi:10.1111/psyp.70050)
Supplement: Supplementary file 1 — Data S1. [file PSYP-62-e70050-s001.docx]

Supplementary Material

| **Table S1** | |
| --- | --- |
| *Maximal (G)LME models for the analysis of behavioral and EEG data* | |
| Analysis | Model formula |
| GLME behavioral analysis |  |
|  | *Accuracy ~ 1 + Feedback Timing * Stimulus Modality * Block + (1 + Feedback Timing * Stimulus Modality * Block \| Subject)* |
| LME N170 analysis |  |
|  | *N170 ~ 1 + Feedback Timing * Feedback Valence * Stimulus Modality * PE * Electrode + (1 + Electrode + Feedback Timing + Stimulus Modality + Feedback Valence + Feedback Timing : Electrode + Feedback Valence : Electrode + Feedback Timing : Stimulus Modality + Feedback Timing : Feedback Valence \| Subject)* |
| LME FRN analysis |  |
|  | *FRN ~ 1 + Feedback Timing * Feedback Valence * Stimulus Modality * PE + (1 + Feedback Timing + Stimulus Modality + Feedback Valence + Feedback Timing : Stimulus Modality + Feedback Timing : Feedback Valence + PE + Feedback Valence : PE \| Subject)* |
| *Note.* GLME = (generalized) linear mixed effects. Feedback Timing (immediate [-0.5] vs. delayed [0.5]), Feedback Valence (negative [-0.5] vs. positive [0.5]), Stimulus Modality (visual [-0.5] vs. auditory [0.5]), PE (scaled and mean centered, yielding negative values for PE values below the mean vs. positive values for PE values above the mean) and Electrode (P7 [-0.5] vs. P8 [0.5]). | |

| **Table S2** | | | | | |
| --- | --- | --- | --- | --- | --- |
| *Results for the GLME analysis on accuracy* | | | | | |
| Effect | β-estimate | *SE* | *z* | *p* |  |
| Block | 1.41 | 0.21 | 6.71 | < .001 | *** |
| Modality | - 0.14 | 0.20 | - 0.69 | .493 |  |
| Timing | 0.16 | 0.11 | 1.46 | .144 |  |
| Block x Modality | - 0.40 | 0.27 | - 1.48 | .140 |  |
| Modality x Timing | 0.25 | 0.25 | 0.99 | .324 |  |
| Block x Timing | 0.18 | 0.21 | 0.84 | .399 |  |
| Block x Modality x Timing | - 0.52 | 0.48 | - 1.08 | .279 |  |
| *Note.* GLME = generalized linear mixed effects, *SE* = standard error, Modality = Stimulus Modality, Timing = Feedback Timing. The sign of the β-estimates indicates the direction of main effects for the fixed-effects predictors Block (1 [-0.5], 2 [-0.167], 3 [0.167], 4 [0.5]), Stimulus Modality (visual [-0.5] vs. auditory [0.5]) and Feedback Timing (immediate [-0.5] vs. delayed [0.5]).  *** *p* < 0.001 | | | | | |

| **Table S3** | | | | | | |
| --- | --- | --- | --- | --- | --- | --- |
| *Results for the LME analysis on the N170 amplitude* | | | | | | |
| Effect | β-estimate | *SE* | *df* | *t* | *p* |  |
| Timing | 0.52 | 0.36 | 38.41 | 1.45 | .156 |  |
| Valence | -0.39 | 0.23 | 45.02 | -1.66 | .104 |  |
| Modality | 0.15 | 0.28 | 41.95 | 0.55 | .583 |  |
| PE | -0.38 | 0.20 | 13196.94 | -1.90 | .057 | . |
| Electrode | -1.67 | 0.58 | 35.55 | -2.85 | .007 | ** |
| Timing x Valence | -0.91 | 0.28 | 68.40 | -3.22 | .002 | ** |
| Timing x Modality | 1.67 | 0.40 | 51.49 | 4.17 | < .001 | *** |
| Valence x Modality | 0.22 | 0.23 | 38090.27 | 0.92 | .356 |  |
| Timing x PE | 0.26 | 0.38 | 1842.60 | 0.69 | .491 |  |
| Valence x PE | -2.18 | 0.42 | 25580.32 | -5.17 | < .001 | *** |
| Modality x PE | 0.85 | 0.38 | 28767.85 | 2.23 | .026 | * |
| Timing x Electrode | 0.33 | 0.44 | 45.89 | 0.76 | .453 |  |
| Valence x Electrode | -0.75 | 0.43 | 47.26 | -1.75 | .087 | . |
| Modality x Electrode | -0.33 | 0.23 | 43198.93 | -1.41 | .159 |  |
| PE x Electrode | -0.28 | 0.40 | 14337.18 | -0.72 | .472 |  |
| Timing x Valence x Modality | 0.35 | 0.46 | 25994.03 | 0.77 | .444 |  |
| Timing x Valence x PE | -1.55 | 0.83 | 15617.27 | -1.86 | .063 | . |
| Timing x Modality x PE | 0.70 | 0.75 | 11251.67 | 0.93 | .351 |  |
| Valence x Modality x PE | 1.23 | 0.82 | 8791.51 | 1.50 | .135 |  |
| Timing x Valence x Electrode | -0.16 | 0.46 | 42974.28 | -0.35 | .730 |  |
| Timing x Modality x Electrode | 1.87 | 0.46 | 41676.48 | 4.03 | < .001 | *** |
| Valence x Modality x Electrode | 0.27 | 0.46 | 41751.62 | 0.59 | .553 |  |
| Timing x PE x Electrode | -1.00 | 0.75 | 42470.22 | -1.35 | .178 |  |
| Valence x PE x Electrode | -2.66 | 0.81 | 41145.40 | -3.28 | .001 | ** |
| Modality x PE x Electrode | -0.16 | 0.75 | 36239.92 | -0.22 | .828 |  |
| Timing x Valence x Modality x PE | 1.57 | 1.64 | 6468.07 | 0.96 | .339 |  |
| Timing x Valence x Modality x Electrode | -0.93 | 0.92 | 43204.28 | -1.01 | .313 |  |
| Timing x Valence x PE x Electrode | -0.35 | 1.59 | 13032.80 | -0.22 | .824 |  |
| Timing x Modality x PE x Electrode | 1.05 | 1.49 | 42818.31 | 0.71 | .480 |  |
| Valence x Modality x PE x Electrode | 0.52 | 1.53 | 42657.98 | 0.34 | .734 |  |
| Timing x Valence x Modality x PE x Electrode | 6.19 | 3.04 | 35302.83 | 2.04 | .042 | * |
| *Note.* LME = linear mixed effects, *SE* = standard error, *df* = degrees of freedom, Timing = Feedback Timing, Valence = Feedback Valence, Modality = Stimulus Modality, PE = PE. The sign of the β-estimates indicates the direction of main effects for the fixed-effects predictors Feedback Timing (immediate [-0.5] vs. delayed [0.5]), Feedback Valence (negative [-0.5] vs. positive [0.5]), Stimulus Modality (visual [-0.5] vs. auditory [0.5]), PE (scaled and mean centered, yielding negative values for PE values below the mean vs. positive values for PE values above the mean) and Electrode (P7 [-0.5] vs. P8 [0.5]).  *** *p* < 0.001, ** *p* < 0.01, * *p* < 0.05, *p* < 0.1 | | | | | | |

| **Table S4** | | | | | | |
| --- | --- | --- | --- | --- | --- | --- |
| *Results of the LME analysis on the FRN amplitude* | | | | | | |
| Effect | β-estimate | *SE* | *df* | *t* | *p* |  |
| Timing | 0.07 | 0.30 | 42.71 | 0.23 | .822 |  |
| Valence | 2.40 | 0.27 | 31.69 | 8.88 | < .001 | *** |
| Modality | 0.62 | 0.24 | 48.93 | 2.55 | .014 | * |
| PE | 0.17 | 0.33 | 22.67 | 0.51 | .613 |  |
| Timing x Valence | -1.07 | 0.38 | 57.33 | -2.85 | .006 | ** |
| Timing x Modality | -1.38 | 0.43 | 54.65 | -3.20 | .002 | ** |
| Valence x Modality | 0.32 | 0.27 | 13460.59 | 1.19 | .236 |  |
| Timing x PE | -0.19 | 0.45 | 2707.83 | -0.43 | .664 |  |
| Valence x PE | 7.44 | 1.01 | 26.42 | 7.36 | < .001 | *** |
| Modality x PE | 0.20 | 0.44 | 10712.29 | 0.46 | .644 |  |
| Timing x Valence x Modality | 0.08 | 0.53 | 16207.28 | 0.16 | .876 |  |
| Timing x Valence x PE | 1.18 | 0.97 | 11120.41 | 1.22 | .222 |  |
| Timing x Modality x PE | -1.09 | 0.87 | 10237.58 | -1.26 | .207 |  |
| Valence x Modality x PE | 1.32 | 0.96 | 6536.76 | 1.37 | .171 |  |
| Timing x Valence x Modality x PE | -2.38 | 1.90 | 6027.52 | -1.25 | .212 |  |
| *Note.* LME = linear mixed effects, *SE* = standard error, *df* = degrees of freedom, Timing = Feedback Timing, Valence = Feedback Valence, Modality = Stimulus Modality, PE = PE. The sign of the β-estimates indicates the direction of main effects for the fixed-effects predictors Feedback Timing (immediate [-0.5] vs. delayed [0.5]), Feedback Valence (negative [-0.5] vs. positive [0.5]), Stimulus Modality (visual [-0.5] vs. auditory [0.5]) and the PE (scaled and mean centered, yielding negative values for PE values below the mean vs. positive values for PE values above the mean).  *** *p* < 0.001, ** *p* < 0.01, * *p* < 0.05 | | | | | | |

| **Figure S1** |
| --- |
| *Descriptive data for the computational prediction error modeling* |
| 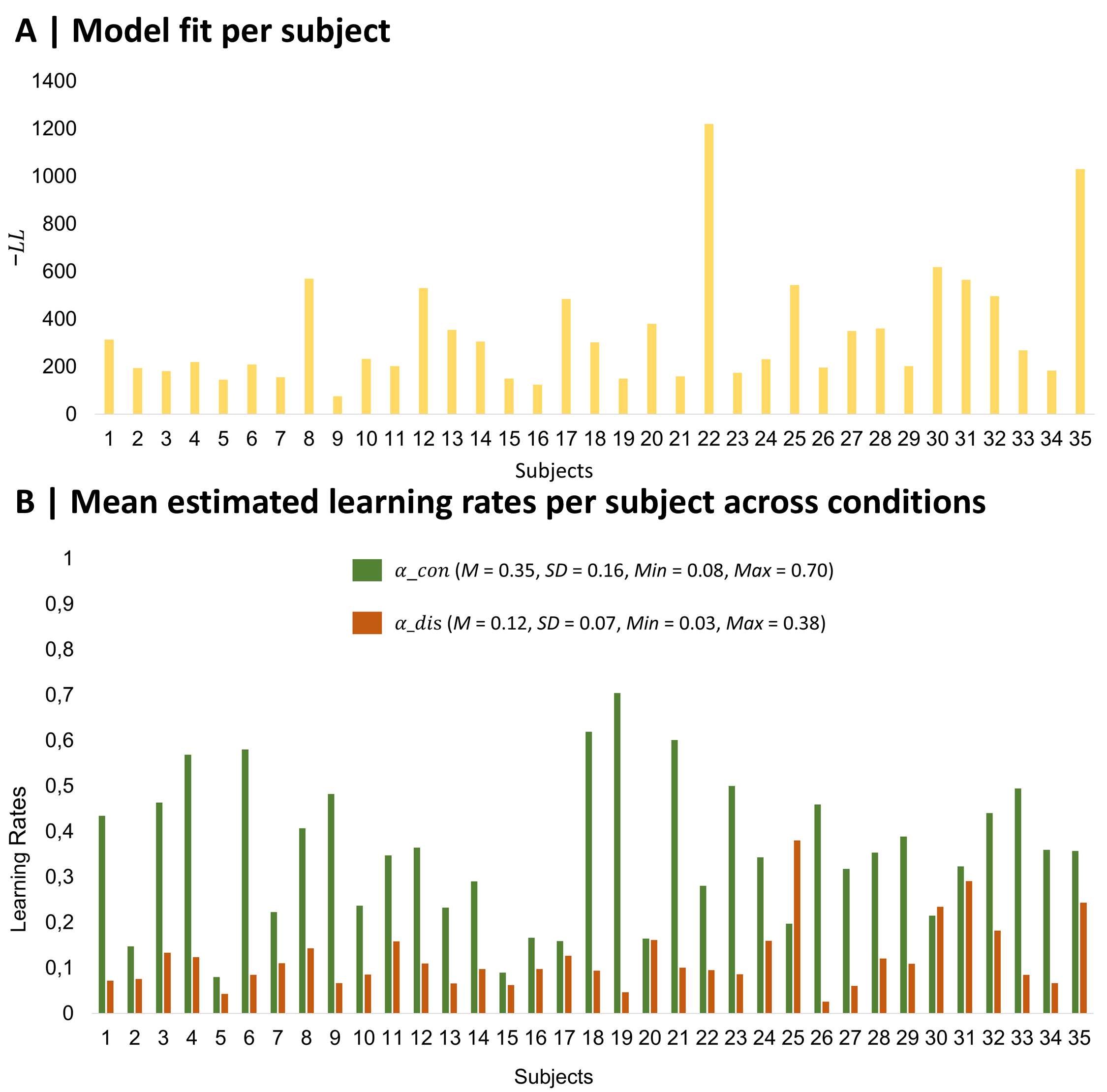 |
| *Note.* **A** Model fit: $-LL$= negative summed log-likelihood as measure for the model’s goodness of fit per subject across conditions. **B** Learning Rates: ­$\alpha_{con}$ = estimated learning rate from positive feedback that confirms the choice, ­$\alpha_{dis}$ = estimated learning rate from negative feedback that disconfirms the choice. |

| **Figure S2** |
| --- |
| *Estimated learning rates from computational prediction error modeling* |
| 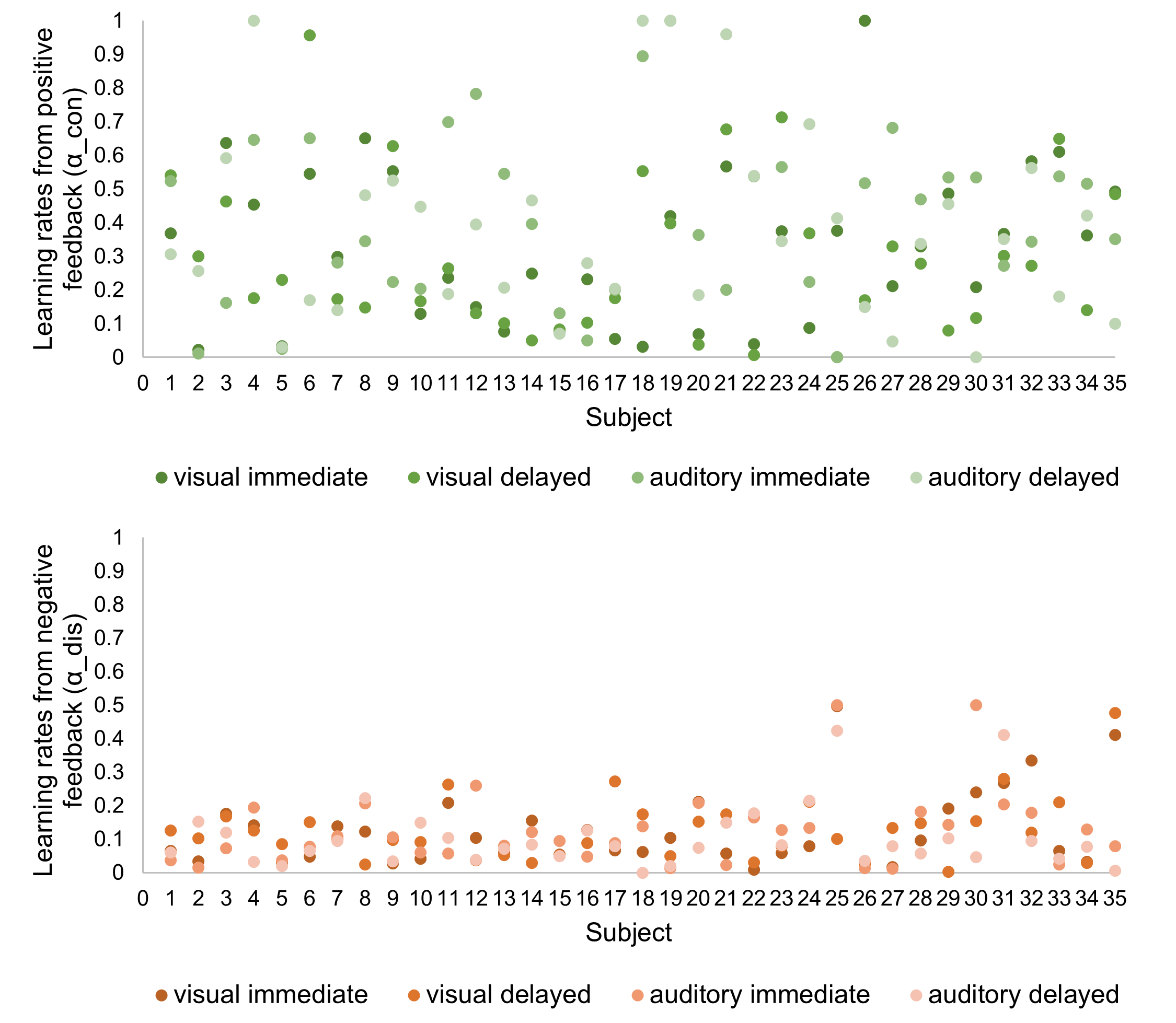 |
| *Note.* Illustrated are the mean learning rates per subject for learning from positive feedback ($\alpha_{con})$ and negative feedback ($\alpha_{dis}$) for the respective Stimulus Modality (visual vs. auditory) and Feedback Timing (immediate vs. delayed) conditions as estimated by the computational prediction error modeling. |

**Win-stay vs. lose-shift analysis**

Our participants generally tended to stick with their previous stimulus choice: In 80% of trials, they selected the same stimulus as in the preceding trial and switched to the alternative stimulus only 20% of the time. Notably, their behavior was influenced by prior feedback: After losing, they maintained their choice in 72.62% of cases, whereas after winning, this percentage increased to 86.28%, which means that they switched more often after a loss or negative feedback. This effect was indeed significant, as revealed by a GLME analysis on the probability of switch responses, including Feedback Valence and absolute PE in the previous trial, as well as their interaction, as fixed factors (random effects were specified as described for the GLME model of the behavioral data in the main text): main effect of Feedback Valence, *z* = -11.23, *p* < .001, *b* = -2.30. There also was a significant main effect of the absolute PE in the previous trial, *z* = -3.68, *p* < .001, *b* = -0.81, which was further explained by a significant interaction between Feedback Valence and absolute PE in the previous trial, *z* = 7,34, *p* < .001, *b* = 14.88. Figure S3 illustrates the underlying pattern in the data: The more unexpected the negative feedback in the previous trial, the greater the tendency of participants to persist with their previous choice. Conversely, the more unexpected positive feedback in the previous trial, the higher the probability of a shift.

| **Figure S3** |
| --- |
| *Win-stay and lose-shift behavior* |
| 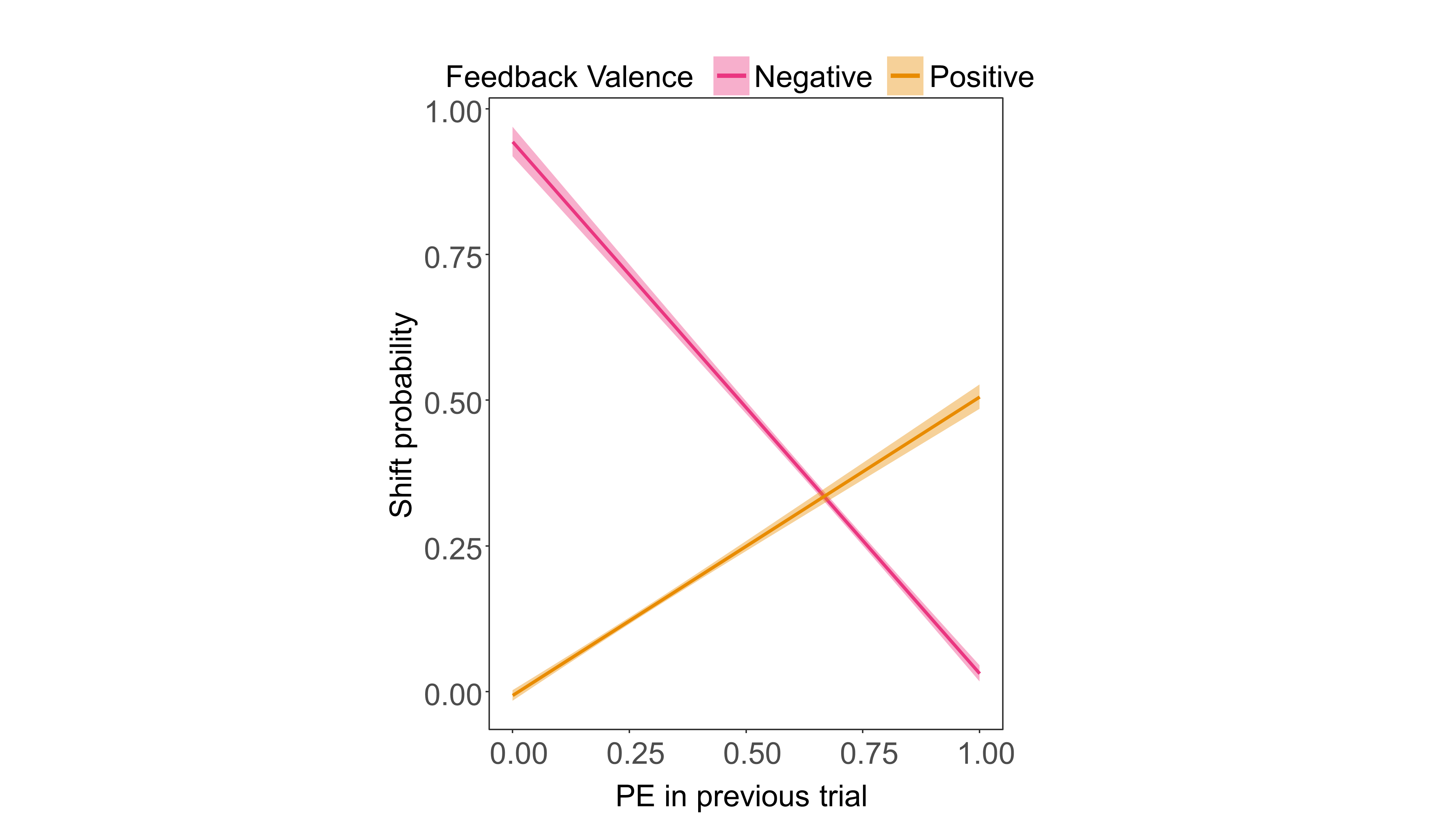 |
| *Note.* PE = prediction error. The y-axis indicates the probability of a shift. A shift value = 0 means that participants stayed with the stimulus they had chosen in the previous trial, a shift value = 1 means that they chose the other stimulus. |

**Figure S4**

*Grand Averages for the N170 following Expected and Unexpected Feedback*

**
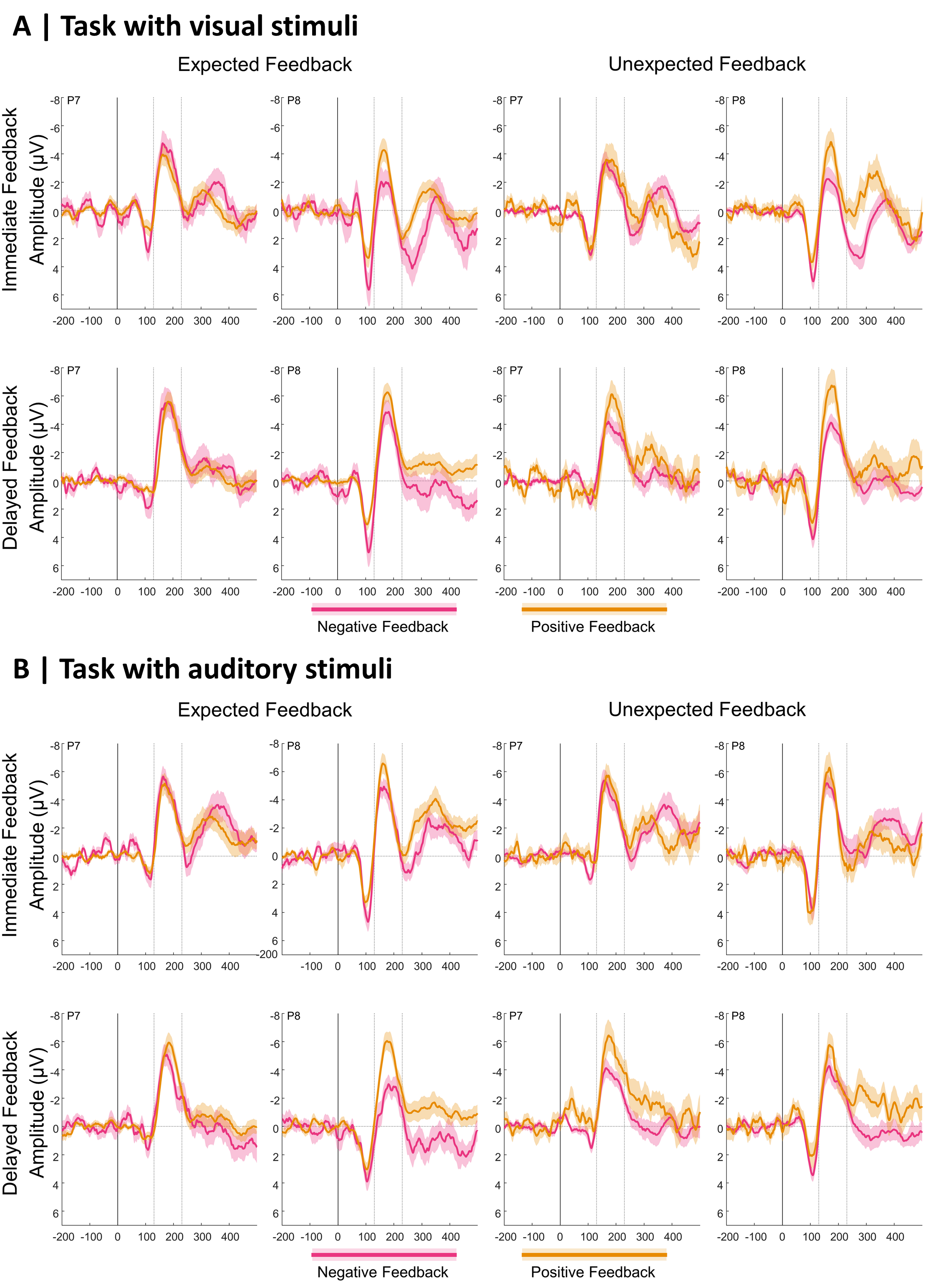
**

*Note.* Dotted lines indicate the time window used for the N170 peak detection. Shaded areas indicate standard errors. Unexpected feedback corresponds to absolute PE values > 0.50, while expected feedback corresponds to absolute PE values ≤ 0.50.

**Figure S5**

*Descriptive data patterns and distributions underlying the N170 analysis*


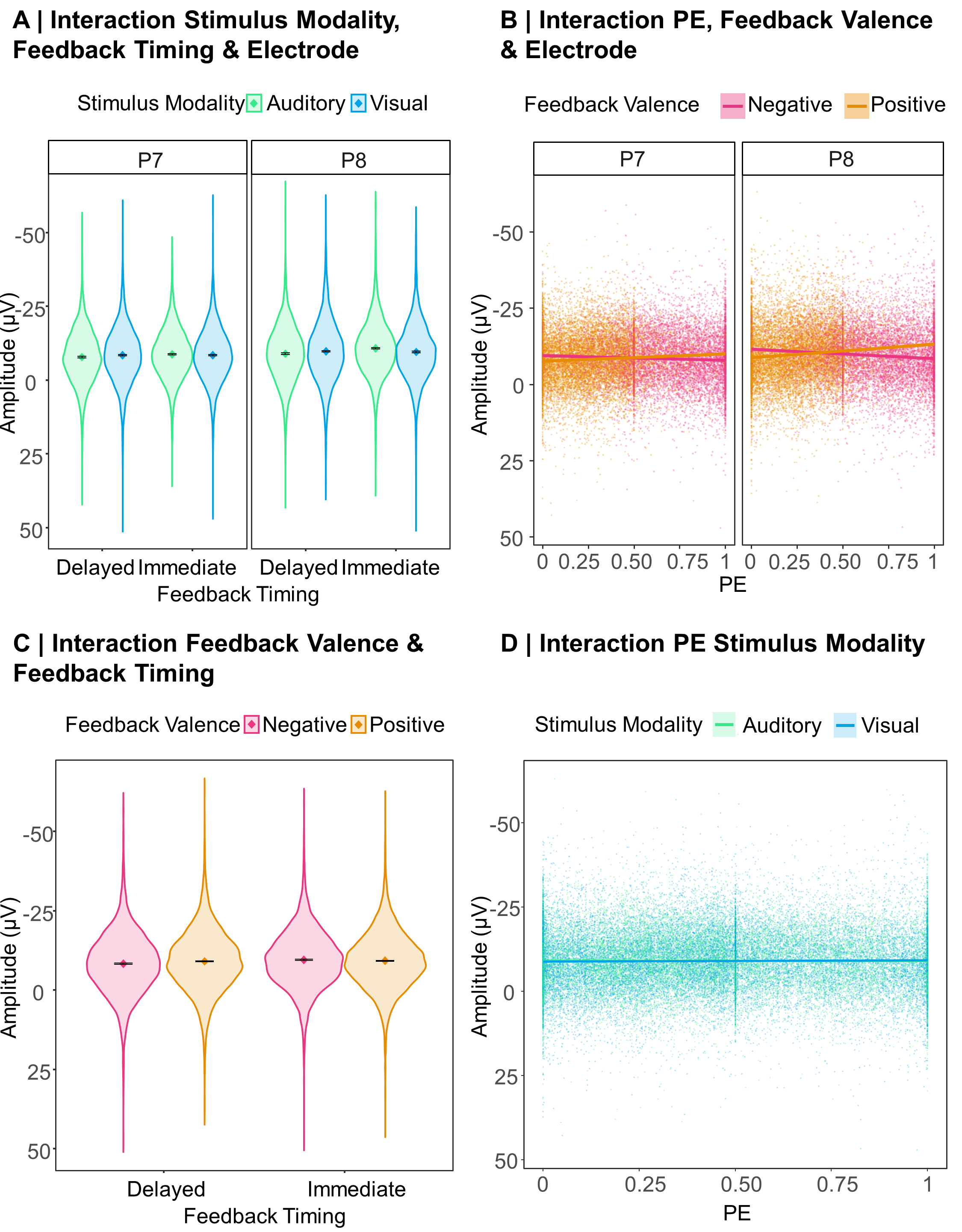


Error bars indicate a 95% confidence interval. Dots represent single-trial data points.

**Figure S6**

*Descriptive data pattern and distribution underlying the PE x Feedback Valence x Modality x Feedback Timing x Electrode interaction for the N170*

*
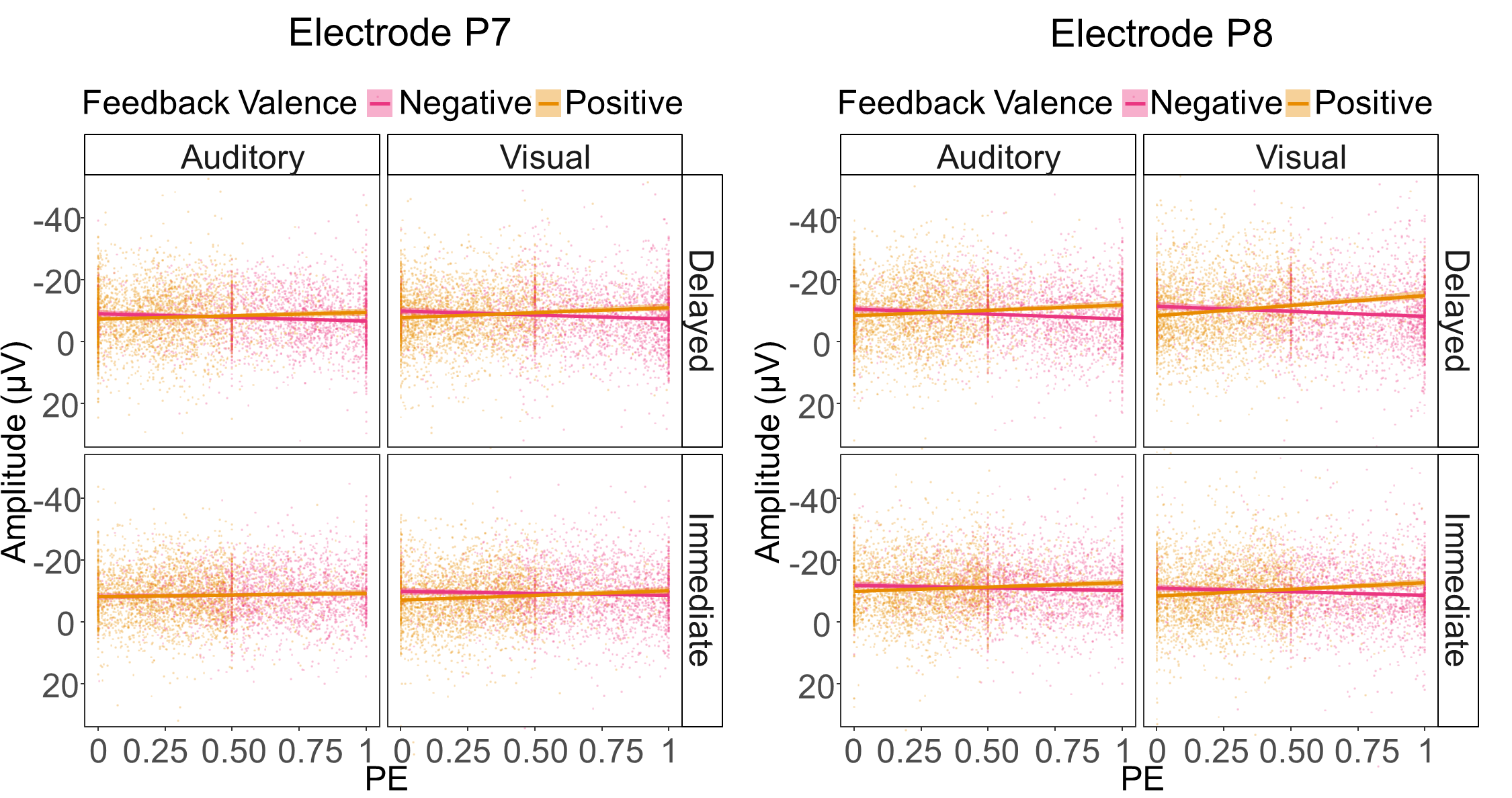
*

Dots represent single-trial data points.

**Figure S7**

*Grand Averages for the FRN following Expected and Unexpected Feedback*

***
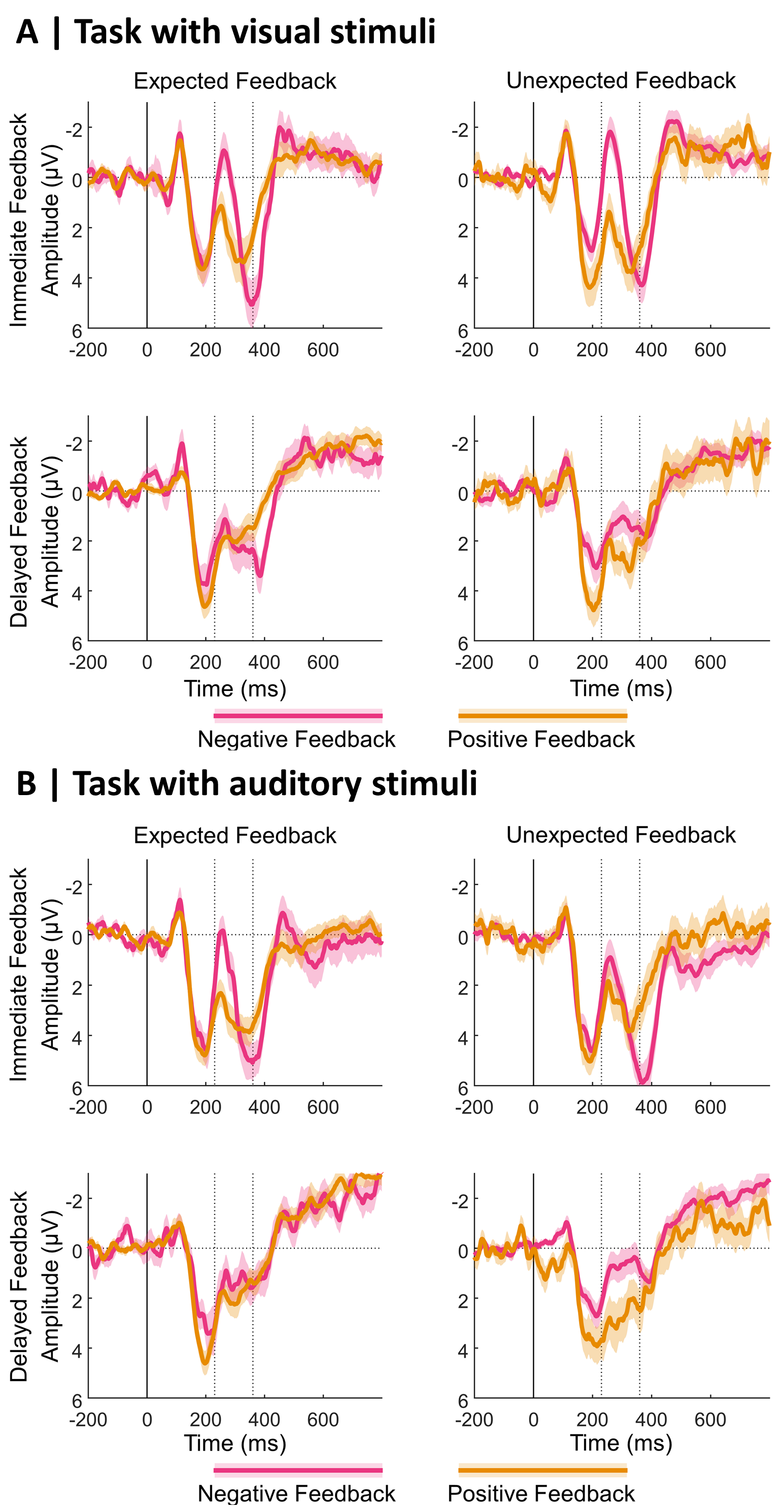
***

*Note.* Dotted lines indicate the time window used for the peak detection in the difference wave (negative – positive feedback). Shaded areas indicate standard errors. Unexpected feedback corresponds to absolute PE values > 0.50, while expected feedback corresponds to absolute PE values ≤ 0.50.

**Figure S8**

*Descriptive data patterns and distributions underlying the FRN analysis*

*
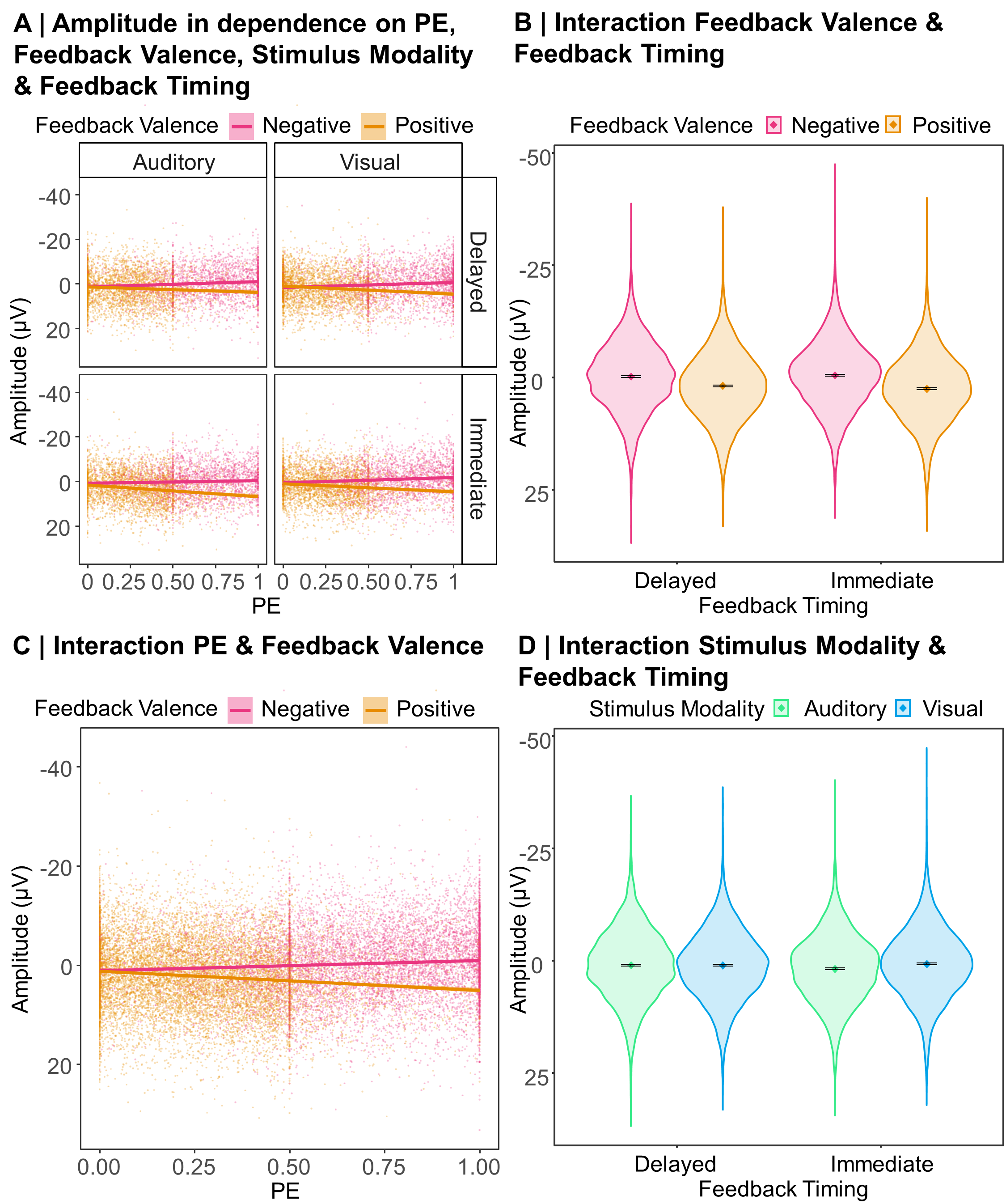
*

Dots represent single-trial data points. Error bars indicate a 95% confidence interval.
